# Impact of Total Indoor Smoking Ban on Cardiovascular Disease Hospitalizations and Mortality: The Case of Chile

**DOI:** 10.1093/ntr/ntae045

**Published:** 2024-03-08

**Authors:** Daniela Montes de Oca, Guillermo Paraje, Cristóbal Cuadrado

**Affiliations:** Universidad Adolfo Ibáñez, Peñalolén, Santiago de Chile, Chile; Business School, Universidad Adolfo Ibáñez, Peñalolén, Santiago de Chile, Chile; Millennium Nucleus for the Evaluation and Analysis of Drug Policies (nDP), Peñalolén, Santiago de Chile, Chile; Millennium Nucleus for the Evaluation and Analysis of Drug Policies (nDP), Peñalolén, Santiago de Chile, Chile; School of Public Health, Universidad de Chile, Santiago de Chile, Chile

## Abstract

**Introduction:**

The harmful effects of first and secondhand smoking are well-established. Smoke-free laws aim at protecting nonsmokers. This study aimed to assess the impact of the 2013 total ban on indoor smoking in Chile on hospitalizations and deaths of major cardiovascular events.

**Aims and Methods:**

The logarithm of the monthly hospitalization and death rates, standardized by age for every 100 000 inhabitants, were estimated for ischemic heart disease, acute myocardial infarction, strokes, and a composite outcome of ischemic heart diseases (which includes acute myocardial infarction) and strokes. In addition, interrupted time series with synthetic control groups were used to assess changes in levels and trends after the intervention.

**Results:**

The total ban on indoor smoking caused significant reductions in death rates for the three diseases studied for age groups above 20 years old. In addition, there were substantial decreases in the post-intervention hospitalization rates for ischemic heart disease: for the 20–44 age group, the decrease was 8.7% compared to the pre-intervention period (*p* < .01). In comparison, such a reduction was 4% (*p* < .01) for the ≥65 age group. For acute myocardial infarction, the decrease was 11.5% (*p* < .01) for the 20–44 age group, while for stroke, it was a 1.2% (*p* < .01) decrease for the total population. It is estimated that the smoking ban averted 15.6% of the deaths compared with the synthetic control groups.

**Conclusions:**

The implementation of total smoke-free environments in Chile contributed to the reduction of mortality for main cardiovascular diseases. This study provides additional evidence of causality linking the policy to health outcomes.

**Implications:**

The total indoor smoking ban significantly affected age-standardized hospitalization and deaths. The number of deaths averted by this policy is estimated at approximately 4758 and 5256 for IHD and stroke, respectively, during the 2013–2017 period (15.6% fewer deaths than predicted by the synthetic control groups). The study contributes to the body of evidence that supports total indoor smoking bans.

## Introduction

It is estimated that approximately 15% of deaths caused by tobacco globally (about 8.1 million deaths per year) occur among nonsmokers (1.2 million deaths per year) as a result of second-hand smoke.^1^ Cardiovascular disease (CVD), including ischemic heart disease (IHD), especially acute myocardial infarction (AMI), and strokes, stand out among the illnesses caused by secondhand smoke.^2^ The harmful effects of secondhand smoke on the cardiovascular system can include a reduction in heart rate variability after moderate exposure for 5–60 min, deterioration in the diastolic function of the left ventricle, reduced tolerance to exercise, and altered vasodilation.^3,4^ In addition, there is solid evidence that smoking cessation quickly reduces some of the negative consequences of prolonged tobacco use.^5,6^

The implementation of smoke-free environments aims to encourage smokers to stop smoking and to protect nonsmokers from harmful exposure to tobacco smoke. Many studies have measured the association between implementing laws on smoke-free environments and CVD hospitalizations and mortality, especially in developed countries. Ireland’s 2004 law immediately reduced death rates from IHD.^7^ Legislation in Helena, Montana, USA, was associated with reducing the total number of hospital admissions for AMI.^8^ The same result was observed in Pueblo, Colorado, USA, after smoke-free environments were implemented in 2003.^9^ Interrupted time series analysis (ITSA) showed that hospitalization rates for AMI fell by 8% after the New York State 2004 total ban on smoking in closed spaces.^10^ In addition, a comparison of the effects of partial and complete bans showed that the total ban was twice as effective as the partial ban. The Arizona 2007 total ban resulted in significant declines in hospitalizations for AMI, unstable angina, and acute strokes in counties without prior legislation.^11^ More recently, a study in Valencia (Spain) found significant reductions in hospital admission rates for CVD, including cerebrovascular events, in the general population following the introduction of a total smoking ban.^12^ Another study conducted for 40 US States found that an increase in the population covered by smoke-free laws is associated with significant decreases in acute cerebrovascular disease hospitalizations among older adults.^13^ A study in Beijing (China) analyzed the effect of a comprehensive set of tobacco control policies, including a total smoking ban, on hospital admissions for all CVD and five major cause-specific CVD.^14^ The authors found significant decreases in hospital admissions of CVD (more than 10%), though they cannot attribute this solely to the smoking ban. All the studies mentioned above do not consider any control group or other robust methods to determine causality and to infer what would have been the counterfactual with no smoke-free bans. Finally, a recent systematic review and meta-analysis on the effect of population-level tobacco control policies found that smoke-free legislation was associated with a statistically significant decrease in the risk for all CVD and respiratory system diseases (RSD) events (including incidence, prevalence, and mortality) and hospitalizations because of both sets of diseases.^15^ The odd ratio for all CVD events was 0.9, for RSD events was 0.83, and for hospitalizations was 0.91 (all significant at 5%).

The body of literature focused on South American countries is limited. After a total ban on indoor smoking in Uruguay in 2006, a reduction of 22% in AMI hospitalizations was observed.^16^ In the same year, in Santa Fe, Argentina, a decrease of 13% in acute coronary syndrome hospitalization was found after a total smoking ban.^17^ The cases of Argentina and Uruguay are peculiar within South America. Uruguay was the first country in the region to implement a total ban in 2006, along with several other measures to curb tobacco consumption (eg, graphic warnings, taxes, etc.).^18^ In Argentina’s case, no national law prohibits consumption in indoor public places. Provinces may have their laws with different provisions. No control group was considered in the studies conducted in Argentina and Uruguay on the effect of smoking bans on health outcomes. Hence, no causality could be attributed to the total ban.

In Chile, a country with one of the highest prevalences of tobacco use globally, tobacco control regulations were implemented at the national level, but gradually.^19^ In 2006, the year when a partial ban on indoor smoke was implemented (with separate spaces for smokers and nonsmokers), the prevalence of tobacco use was 42.4%.^20^ Legislation implementing a total ban on indoor smoking was enacted in 2013.^21^ Before–after analysis using data from 2011 to 2014 found that this policy almost immediately affected the incidence of AMI.^22^ No effect was found on total ischemic stroke events. However, no causality could be established because of the analysis’s lack of a control group. In mid-2005, the Explicit Health Guarantees (GES) was implemented. The GES program prioritizes providing healthcare with a pre-defined list of conditions/diseases, with national clinical guidelines, maximum waiting times, financial protection to patients, etc.^23^ AMI and strokes were among the first conditions to be covered by the GES.^24^

This study aimed to assess the effect of the 2013 total ban on indoor smoking in Chile on hospitalizations and deaths because of IHD, in general, AMI, in particular, and strokes, for the entire population (ages 20 years old and more) and several specific age groups. In addition, two analyses are also included: (i) hospitalizations and deaths for a composite outcome of ischemic heart disease (which includes AMI) and stroke and (ii) hospitalization and death rates by sex and age groups. These additional results are mentioned in the manuscript, and the tables are available in the Supplementary Material. Logarithms of monthly hospitalization and death rates between 2007 and 2017, standardized by age for every 100 000 inhabitants, were used. As an innovation from previous studies in Chile and other countries, synthetic control groups that allow causality to be attributed to the smoke-free environment regulation were used.

## Methods

### Study Population

This study used daily data, between 1 January 2007 and 31 December 2017, from two databases from the Chilean Ministry of Health: (1) the database of hospital discharges and (2) the database of deaths.^25,26^ The hospital discharge database includes national discharges from private and public hospitals and healthcare facilities. Likewise, the death database registers all deaths registered in the country, both in-patients and outside healthcare facilities. Data quality for mortality statistics based on death certificates in Chile is considered high by international standards, with coverage above 99.7%.^27^ Hospital discharge databases include 98% of all healthcare centers that provide inpatient care in the country (*n* = 328), including public and private providers.^28^ Both databases include the primary hospitalization and death diagnoses according to the International Statistical Classification of Diseases and Related Health Problems (ICD-10).^7,29^ The codes used for IHDs are I20–I25, which include AMI (I21) and angina (I20), and the codes for strokes are I61–I64 and I67. First events and rehospitalizations are not separated in the analyses, as most observations are first events (for instance, 88.4% of AMI and 72.1% of strokes). Analyses conducted only with the first events show no significant difference from those presented here.

Databases are public and contain anonymized data, so no Ethics Committee approval was needed. In this study, neither patients nor the public were involved in any way at any stage.

### Statistical Analysis

Daily events were aggregated monthly by 5-year age groups for each identified disease. The logarithm of specific rates was estimated by age using annual national population information by age group and standardized population rates using World Health Organization population weightings.^30,31^ Using an international standard population allows comparisons with other countries and/or periods. As a result, a database with monthly observations (2007–2017) of the logarithm of hospitalization and death rates standardized by age (20–44, 45–64, and ≥ 65 years, and the total population aged >20 years) for the diseases mentioned above was obtained. Events for the population below 20 years old were not considered, as the incidence rates for hospitalizations and deaths from tobacco-related diseases for such a population are negligible.

The unit of analysis for this method is time rather than individuals. Consequently, ITSA models with a synthetic control group were used for the estimates.^32,33^ The ITSA model with a control group and one intervention (the total ban on indoor smoking) is as follows:


$$\begin{array}{lll} Y_{t}= & \;\beta_{0}+\beta_{1}T_{t}+\beta_{2}X_{t}+\beta_{3}X_{t}T_{t}+\beta_{4}Z\; & +\beta_{5}ZT_{t}+\beta_{6}ZX_{t}+\beta_{7}ZX_{t}T_{t}+\epsilon_{t}\; \end{array}$$

where $Y_{t}$ is the logarithm of the standardized monthly rates of hospitalizations or deaths from the respective diseases and age groups; $T_{t}$ is a linear time trend (in months); $X_{t}$ is a dichotomous variable indicating the intervention’s implementation (equal to 1 as of February 2014, see below); *Z* is a dichotomous variable indicating whether it is the treatment group, or the control group (equal to 1 if it is the treatment group).^34^ Interpretation of parameters has been thoroughly described elsewhere and is mentioned in the Supplementary Material.^32,34,35^ The most relevant ones are: $\beta_{4}$ is the difference in levels between the treated and control groups in the first observation of the pre-intervention period; $\beta_{5}$ is the difference in the pre-intervention trends between the treated and control groups; $\beta_{6}$ is the difference in levels between the pre and postintervention periods and between the treated and control groups; and *β*_7_ is the difference in trends between the pre- and postintervention periods and between the treated and control groups. Nonsignificant $\beta_{4}$ and $\beta_{5}$ parameters imply that control and treatment groups have similar statistical behaviors pre-intervention. In comparison, significant $\beta_{6}$ and $\beta_{7}$ parameters imply that the levels and trends of both groups drift apart after the intervention, respectively.

Synthetic control groups were constructed for each condition, sex, and age group from a donor pool of hospitalizations and deaths from diseases unrelated to tobacco use for each sex and age group. Diseases covered at any time by the GES program were not considered in the donor pool. The list of the 33 diseases/conditions in the donor pool is in the Annexure in the Supplementary Material.

Observations were chosen and weighted so synthetic control groups replicate the treatment group’s statistical behavior (in levels and trends) pre-intervention. In such a case, the synthetic control group is the counterfactual to how the treatment group would have behaved without the intervention. The Synthetic Control Using Lasso (SCUL) method was used to construct synthetic control groups.^36^ Placebo tests for the control groups are presented in the Methodological Annexure in the Supplementary Material (Tables A1 and A2 and Figures A1 and A2).^37^

Because mortality time series are highly seasonal, annual moving averages were used. Thus, the observation for a given month is the simple average for that month and the 11 previous ones. Consequently, February 2014 was considered the start of the intervention because it is the first full year of months with the total smoking ban (including March 2013, the actual date of the intervention, and the 11 months that followed). The models were estimated using OLS with errors corrected by applying Newey-West to correct autocorrelation and heteroskedasticity.^38^ Given that this study sought to determine whether hospitalization and death rates declined with the implementation of the total ban, one-tail significance tests were used for the significance of the coefficients (full tables with results are presented in the Supplementary Material). Results for males are presented in Tables S9–S16, while those for females are presented in Tables S17–S24. The usual two-tailed significance tests for the main results are shown in Tables S25–S32.

## Results

In Table 1, Part A shows the mean values and corresponding confidence intervals for pre- and post-intervention age-standardized hospitalization rates (per 100 000 inhabitants). IHD and strokes post-intervention means were lower than the pre-intervention period, while the reverse was observed for AMI in all cases.

**Table 1.** Mean and Confidence Intervals of the Pre-intervention and Post-intervention Age-standardized Hospitalization and Death Rates per 100 000 Inhabitants

Part A: Age-standardized hospitalization rates per 100 000 inhabitants								
	Ischemic heart disease		AMI		Stroke		Composite outcome	
	Pre-intervention	Post-intervention	Pre-intervention	Post-intervention	Pre-intervention	Post-intervention	Pre-intervention	Post-intervention
	20–44 years							
Mean	1.784	1.615	0.792	0.89	1.767	2.024	3.552	3.64
95% CI	[1.772, 1.796]	[1.601, 1.63]	[0.776, 0.808]	[0.869, 0.91]	[1.737, 1.797]	[2.015, 2.033]	[3.522, 3.581]	[3.629, 3.65]
	45–64 years							
Mean	21.53	19.789	8.511	9.631	15.084	15.02	36.614	34.808
95% CI	[21.285, 21.775]	[19.72, 19.858]	[8.411, 8.611]	[9.502, 9.76]	[14.986, 15.182]	[14.911, 15.128]	[36.438, 36.79]	[34.675, 34.942]
	≥65 years							
Mean	53.838	48.99	20.137	22.092	73.093	66.858	126.932	115.848
95% CI	[53.461, 5 4.216]	[48.631, 49.348]	[19.838, 20.435]	[21.796, 22.388]	[72.861, 73.326]	[66.059, 67.656]	[126.385, 127.478]	[114.801, 116.894]
	Entire population							
Mean	9.364	8.553	3.634	4.053	9.758	9.333	19.123	17.885
95% CI	[9.285, 9.444]	[8.515, 8.59]	[3.584, 3.683]	[3.999, 4.107]	[9.735, 9.782]	[9.248, 9.418]	[19.058, 19.188]	[17.781, 17.989]
Part B: Age-standardized death rates per 100 000 inhabitants								
	Ischemic heart disease		AMI		Stroke		Composite outcome	
	Pre-intervention	Post-intervention	Pre-intervention	Post-intervention	Pre-intervention	Post-intervention	Pre-intervention	Post-intervention
	20–44 years							
Mean	0.265	0.263	0.232	0.22	0.189	0.148	0.455	0.411
95% CI	[0.262, 0.269]	[0.257, 0.27]	[0.229, 0.235]	[0.216, 0.224]	[0.186, 0.192]	[0.142, 0.153]	[0.449, 0.46]	[0.402, 0.42]
	45–64 years							
Mean	3.895	3.635	3.199	2.925	2.234	1.783	6.129	5.419
95% CI	[3.837, 3.953]	[3.572, 3.698]	[3.158, 3.239]	[2.875, 2.976]	[2.199, 2.27]	[1.737, 1.829]	[6.038, 6.22]	[5.312, 5.525]
	≥65 years							
Mean	27.01	23.242	19.992	17.171	23.242	18.103	50.252	41.345
95% CI	[26.389, 27.631]	[23.001, 23.483]	[19.586, 20.397]	[17.004, 17.338]	[22.831, 23.654]	[17.659, 18.547]	[49.235, 51.269]	[40.693, 41.997]
	Entire population							
Mean	3.092	2.729	2.364	2.073	2.431	1.903	5.522	4.632
95% CI	[3.03, 3.153]	[2.696, 2.763]	[2.324, 2.404]	[2.05, 2.097]	[2.389, 2.472]	[1.856, 1.95]	[5.421, 5.624]	[4.555, 4.71]

AMI = acute myocardial infarction; CI = confidence interval.

In Table 1, Part B shows the mean values and corresponding confidence intervals for pre- and post-intervention age-standardized death rates (per 100 000 inhabitants). In all age groups for IHD, AMI, and stroke, the post-intervention rates were lower than the pre-intervention ones.

Table 2 shows the effect of the total ban on indoor smoking on monthly hospitalizations for IHDs, AMIs, strokes (Figures 1–3 in the Supplementary Material, respectively), and the composite outcome. Again, the synthetic control groups created for each age group and the total population replicated the behavior of the treatment group in the pre-intervention period (both $\beta_{4}$ and $\beta_{5}$ were statistically nonsignificant in all cases, except for stroke hospitalizations for the ≥65 years old group, and for the composite outcome for the entire population, the 45–64, and the ≥65 years old group, as shown in Tables S1–S3).

**Table 2.** Estimated Effect on the Logarithm of the Monthly Age-standardized Hospitalization Rates for Ischemic Heart Disease, Acute Myocardial Infarction, and Stroke per 100 000 Inhabitants (Period 2007–-2017) (One-tail Significance Test)

		Effect on level of intervention (β6)	Effect on trend of intervention (β7)
		Log(rate)	Log(rate)
Ischemic heart disease	Entire population		
	Coefficient	−0.001	−0.000
	95% IC	[−0.031, 0.029]	[−0.001, 0.000]
	20–44 years		
	Coefficient	−0.087***	0.001
	95% IC	[−0.137, −0.038]	[−0.001, 0.002]
	45–64 years		
	Coefficient	−0.015	0.003
	95% IC	[−0.041, 0.011]	[0.002, 0.004]
	≥65 years		
	Coefficient	−0.040**	0.002
	95% IC	[−0.079, −0.001]	[0.001, 0.004]
Acute myocardial infarction	Entire population		
	Coefficient	0.001	0.000
	95% IC	[−0.028, 0.030]	[−0.001, 0.001]
	20–44 years		
	Coefficient	−0.115***	0.005
	95% IC	[−0.151, −0.080]	[0.004, 0.007]
	45–64 years		
	Coefficient	−0.017**	0.000
	95% IC	[−0.037, 0.002]	[−0.001, 0.001]
	≥65 years		
	Coefficient	0.023	0.002
	95% IC	[−0.037, 0.083]	[−0.000, 0.003]
Stroke	Entire population		
	Coefficient	−0.012***	−0.002***
	95% IC	[−0.020, −0.003]	[−0.003, −0.002]
	20–44 years		
	Coefficient	0.044	−0.001*
	95% IC	[0.024, 0.063]	[−0.001, 0.000]
	45–64 years		
	Coefficient	0.011	−0.004***
	95% IC	[−0.014, 0.035]	[−0.005, −0.003]
	≥65 years		
	Coefficient	−0.013	−0.003
	95% IC		
Composite outcome	Entire population		
	Coefficient	−0.013	−0.001***
	95% IC	[−0.034, 0.008]	[−0.001, −0.000]
	20–44 years		
	Coefficient	−0.025*	0.000
	95% IC	[−0.058, 0.008]	[−0.000, 0.001]
	45–64 years		
	Coefficient	−0.012	0.001
	95% IC	[−0.030, 0.007]	[−0.000, 0.001]
	≥65 years		
	Coefficient	−0.018*	−0.001***
	95% IC	[−0.040, 0.004]	[−0.002, −0.001]

***p < .01; **p < .05; *p < .1.

In the case of IHDs, across all age groups, except for the total population, the intervention had an immediate (level) effect on the logarithm of rates of total monthly hospitalizations per 100 000 inhabitants. Precisely, $\beta_{6}$ estimated that the intervention was associated with a −0.14 reduction (*p* < .01) in hospitalizations per 100 000 inhabitants in the 20–44 years age group (an 8.7% drop compared with the logarithm of the pre-intervention rates). For the ≥65 years age group, the reduction was −1.99 (*p* < .05) total events per 100 000 inhabitants (a 4% drop compared with the logarithm of the pre-intervention rates).

In the case of AMIs, a statistically significant drop (−0.10) in the immediate level of hospitalizations per 100 000 inhabitants was observed (*p* < .01) in the 20–44 years age group. This represents an 11.5% decrease in hospitalizations. An appropriate control group for strokes could not be found for the ≥65 age group. The 45–64 age group showed a 0.07 drop in the trend of monthly hospitalizations for strokes, representing 0.4% of monthly hospitalizations (*p* < .01). The entire population saw an immediate reduction of 1.2% and a decrease of −0.2% in the trend of monthly hospitalizations (both *p* < .01).

Finally, no suitable control groups for the composite outcome were found except for the 20–44 age group. No statistically significant effect of the intervention is found for such a group.

Table 3 shows the effect on deaths from IHDs, AMIs, strokes, and the composite outcome. Control groups are appropriate for all groups (both $\beta_{4}$ and $\beta_{5}$ were statistically nonsignificant in all cases except for the stroke death rates in the 20–44 age groups, as shown in Tables S5–S8). As expected, no immediate effect is observable on IHD deaths. Nevertheless, a consistent reduction in deaths is observed through the change in trends in the different age groups. There was a −0.6% change in the logarithm of the monthly death rate in the 20–44 age group; 45–64 years, −0.5%; ≥65 years, −0.3%; and the entire population, −0.5% (Figure S4).

**Table 3.** Estimated Effect on the Logarithm of the Monthly Age-standardized Death Rates for Ischemic Heart Disease, Acute Myocardial Infarction, and Stroke per 100 000 Inhabitants (Period 2007–2017) (One-tail Significance Test)

		Effect on level of intervention (β6)	Effect on trend of intervention (β7)
		Log(rate)	Log(rate)
Ischemic heart disease	Entire population		
	Coefficient	0.086	−0.005***
	95% IC	[0.021, 0.150]	[−0.007, −0.003]
	20–44 years		
	Coefficient	0.076	−0.006***
	95% IC	[−0.008, 0.160]	[−0.009, −0.003]
	45–64 years		
	Coefficient	0.081	−0.005***
	95% IC	[0.018, 0.144]	[−0.007, −0.004]
	≥65 years		
	Coefficient	0.138	−0.003**
	95% IC	[0.045, 0.231]	[−0.007, −0.000]
Acute myocardial infarction	Entire population		
	Coefficient	0.027	−0.004***
	95% IC	[−0.020, 0.073]	[−0.006, −0.003]
	20–44 years		
	Coefficient	−0.015	−0.005***
	95% IC	[−0.050, 0.021]	[−0.007, −0.004]
	45–64 years		
	Coefficient	0.056	−0.005***
	95% IC	[0.021, 0.092]	[−0.006, −0.004]
	≥65 years		
	Coefficient	0.098	−0.004***
	95% IC	[0.021, 0.174]	[−0.007, −0.002]
Stroke	Entire population		
	Coefficient	0.070	−0.008***
	95% IC	[0.035, 0.105]	[−0.009, −0.006]
	20–44 years		
	Coefficient	−0.077**	−0.005***
	95% IC	[−0.166, 0.011]	[−0.009, −0.001]
	45–64 years		
	Coefficient	0.007	−0.009***
	95% IC	[−0.028, 0.042]	[−0.010, −0.008]
	≥65 years		
	Coefficient	0.108	−0.009***
	95% IC	[0.064, 0.152]	[−0.011, −0.008]
Composite outcome	Entire population		
	Coefficient	0.075	−0.006***
	95% IC	[0.026, 0.123]	[−0.008, −0.004]
	20–44 years		
	Coefficient	0.024	−0.005***
	95% IC	[−0.021, 0.069]	[−0.006, −0.003]
	45–64 years		
	Coefficient	0.055	−0.007***
	95% IC	[0.004, 0.107]	[−0.008, −0.005]
	≥65 years		
	Coefficient	0.120	−0.006***
	95% IC	[0.048, 0.191]	[−0.009, −0.004]

***p < .01; **p < .05.

Consistent with IHD, a change in the mortality trend for AMIs is observed after policy enforcement (Figure S5). The drop in monthly deaths was 0.5% (*p* < .01) in the 20–44 and 45–64 years age groups and 0.4% (*p* < .01) among those aged ≥65 years and in the entire population.

Concordant with the observed reduction in the deaths for IHD and AMI, the stroke mortality trend also reduced after the ban (Figure S6). The drop in monthly deaths in the group aged 45–64 years was 0.9% (*p* < .01), 0.9% (*p* < .01) for the population ≥65 years, and 0.8% (*p* < .01) for the entire population.

Finally, the composite outcome shows that the trend in monthly deaths decreased for all age groups and the total population. Such a decrease ranged from 0.5% (*p* < .01) for the 20–44 group to 0.7% (*p* < .01) for the 45–64 years group.

The logarithm of the hospitalization rates for IHD did not show a significant decrease in trend in either sex. The 8.7% decrease in level found in the logarithm of the hospitalization rates for ischemic heart disease in the group 20**–**44 years old is attributable to males (Table S9), as they presented a 13.0% decrease in the logarithm of the hospitalization rates level after the intervention. Similarly, the change in the levels of hospitalization rates for males explains the changes found for the entire population for AMI hospitalizations (Table S10). The logarithm of the stroke hospitalization rates shows statistically significant decreases in trends for the age groups 45-64 years, ≥65 years, and the total population for both men and women (see Tables S11 and S19, respectively), as well as the general population (Table 2).

The trends of the logarithm of the IHD death rates for males show statistically significant declines in all age groups, in contrast to females, who only show statistically significant decreases in the 45–64 age group (Tables S13 and S21, respectively). The total population shows a decreasing trend in all age groups except for those aged 65 and older (Table 3). The trend of the logarithm of the AMI death rates decreased statistically significantly for all age groups of the total population (Table 3), which was primarily explained by similar decreases in the male population (Table S14). Finally, the trends of the logarithm of death rates for stroke show a statistically significant reduction for all age groups of females (Table S23) and the total population (Table 3). In contrast, a significant decrease was observed for males aged 45–64, 65 and over, and the entire male population (Table S15).

## Discussion

Total bans on indoor smoking can influence health outcomes through various mechanisms. First, active smokers are strongly discouraged from smoking, requiring them to move to designated areas for smoking.^39^ Additionally, total bans increase cessation rates and quit attempts among active smokers.^40,41^ Second, for the rest of the population, passive exposure to smoking is drastically reduced in closed environments, including restaurants, bars, workspaces, etc., in which the persons experience a significant potential exposure, even when a single person is smoking. Both first and second-hand smoke exposures are well-established risk factors for cardiovascular events.^42,43^ Therefore, the indoor smoking ban acts as a population-level intervention reducing exposure to harmful substances with potentially positive effects on CVD incidence and mortality for every person in the territory that enacted the policy. The dataset we have does not allow us to test which of these different channels prevailed, as the smoking status of people suffering from CVD is not recorded.

Comparing the decreases in smoking prevalence pre- and post-total ban can give very indirect evidence on which channel may prevail. From 2006 to 2012, the decrease in smoking prevalence in the 12–64 population was 19.8%, while the decrease was 10.4% between 2014 and 2018.^44^ This might indicate that the reduction in CVD events could have come from a reduction in SHS exposure. However, this conclusion must be considered with extreme care, as it is based on indirect data.

The results show that Chile’s total ban on indoor smoking had sizable and sustained effects, reducing hospitalizations and deaths from IHD in general and AMI and strokes in particular. The decline in deaths from strokes in the entire population is particularly noteworthy. These results are consistent with those of studies performed in other countries (with different levels of coverage of the measure) and periods.^7,8,45–47^ These results are also consistent with those of a previous study conducted in Chile. However, the results from this study include comparisons with a synthetic control group, which increases their robustness and provides additional evidence for causality.^22^

AMI and strokes (in people aged ≥15 years) were included in a list of priority care diseases (GES) with national clinical guidelines, maximum waiting times, and specific financial protection since 2005. This policy can explain the reduced mortality attributable to them in the pre-intervention period.^24^ This can be seen in the results obtained based on a downward trend in the logarithm of the pre-intervention period for the stroke mortality rate for the total population, for example, and to a lesser extent for AMI for the entire population. However, as our analyses only considered the period after their inclusion in the GES, the effect of this policy was controlled. No major change in the tobacco control policy occurred around the time or immediately after the implementation of the total indoor ban that could have affected CVD hospitalizations and deaths.

In the case of hospitalizations, the largest effects were found in the 20–44 years group. Younger groups are likely more exposed to second-hand smoke in bars, restaurants, clubs, etc., and this could explain why the smoking ban had greater impacts on CVD in this age group. However, we did not have data on SHS exposure by age group, so this hypothesis could not be tested in this study.

The number of deaths averted by this policy could be estimated using the $\beta_{7}$ coefficients for IHD and strokes (all significant at 1%) in the entire population. Comparing the number of deaths predicted in the synthetic control group with the actual number of deaths, it is estimated that approximately 4758 and 5256 deaths from IHD and stroke, respectively, were averted in the analyzed period. This would imply 15.6% fewer deaths than predicted by the control group.

The analysis performed here has some limitations. First, the differential effect of the 2013 regulation establishing a total ban on indoor smoking and the 2006 partial ban could not be measured. Some articles have found that a complete ban significantly affects CVD more than a partial ban.^10^ In the case of Chile, the effect of the partial ban and the GES program, which began in 2006 and included AMI and strokes, cannot be separated. Second, there was no information on the inhabitants’ smoking status (smokers/nonsmokers). This prevents measuring differential effects of the ban between groups^47,48^ or estimating the impact of the regulation on smoking cessation.

Policies on totally smoke-free environments, one of the central policies in tobacco control, have almost immediate effects on the population’s health. This is because exposure to secondhand smoke has numerous well-documented adverse effects, and eliminating that exposure leads to health gains in the population. This study on the indoor smoking ban in Chile provides evidence and allows linking this policy to the health results using synthetic control groups.

## Conclusions

Total smoke-free environments, one of the central policies in tobacco control, may have almost immediate effects on the population’s health. This study’s methodology strengthened previous results, mainly from developed countries, by using a synthetic control group, which provides additional evidence for causality.

## Supplementary Material

ntae045_suppl_Supplementary_Material

## Data Availability

The data underlying this article are available in DEIS (Department of Statistics and health information, Ministry of Health) at https://deis.minsal.cl/#datosabiertos.
