# Supplementary material for: Impact of Total Indoor Smoking Ban on Cardiovascular Disease Hospitalizations and Mortality: The Case of Chile
Source: Nicotine Tob Res. 2024 Mar 8;26(9):1166–74. doi: 10.1093/ntr/ntae045 (PMC11339173; doi:10.1093/ntr/ntae045)
Supplement: ntae045_suppl_Supplementary_Material [file ntae045_suppl_supplementary_material.docx]

**Supplemental material**

Figure S1. Monthly age-standardized hospitalization rates for ischemic heart diseases for 2007–2017.

Figure S2. Monthly age-standardized hospitalization rates for acute myocardial infarction from 2007–2017.

Figure S3. Monthly age-standardized hospitalization rates for strokes for the period 2007–2017.

Figure S4. Monthly age-standardized death rates for ischemic heart diseases for 2007–2017.

Figure S5: Monthly age-standardized death rates for acute myocardial infarction from 2007–2017.

Figure S6. Monthly age-standardized death rates for strokes for the period 2007–2017.

Table S1: Estimated effect on monthly age-standardized hospitalization rates for ischemic heart disease per 100,000 inhabitants (period 2007-2017) (One-tail significance test)

|  | 20-44 | 45-64 | 65+ | Entire population |
| --- | --- | --- | --- | --- |
|  | Log(rate) | Log(rate) | Log(rate) | Log(rate) |
| Initial control trend ($\beta_{1}$) |  |  |  |  |
| Coefficient | -0.001*** | -0.002*** | -0.001*** | -0.002*** |
| 95% CI | [-0.001,-0.000] | [-0.002,-0.002] | [-0.001,-0.001] | [-0.002,-0.001] |
| Difference in initial level difference between treated and control ($\beta_{4}$) |  |  |  |  |
| Coefficient | 0.000 | 0.003 | -0.000 | 0.000 |
| 95% CI | [-0.020, 0.020] | [-0.015, 0.021] | [-0.028, 0.027] | [-0.017, 0.018] |
| Difference in pre-intervention trend between treated and control ($\beta_{5}$) |  |  |  |  |
| Coefficient | -0.000 | -0.000 | -0.000 | -0.000 |
| 95% CI | [-0.001, 0.000] | [-0.001, 0.000] | [-0.001, 0.001] | [-0.000, 0.000] |
| Immediate effect (level) of intervention ($\beta_{6}$) |  |  |  |  |
| Coefficient | -0.087*** | -0.015 | -0.040** | -0.001 |
| 95% CI | [-0.137,-0.038] | [-0.041, 0.011] | [-0.079,-0.001] | [-0.031, 0.029] |
| Effect on trend of intervention ($\beta_{7}$) |  |  |  |  |
| Coefficient | 0.001 | 0.003 | 0.002 | -0.000 |
| 95% CI | [-0.001, 0.002] | [0.002, 0.004] | [0.001, 0.004] | [-0.001, 0.000] |

*** p<0.01; ** p<0.05; * p<0.1

Table S2: Estimated effect on monthly age-standardized hospitalization rates for AMI per 100,000 inhabitants (period 2007-2017) (One-tail significance test)

|  | 20-44 | 45-64 | 65+ | Entire population |
| --- | --- | --- | --- | --- |
|  | Log(rate) | Log(rate) | Log(rate) | Log(rate) |
| Initial control trend ($\beta_{1}$) |  |  |  |  |
| Coefficient | 0.004 | 0.002 | 0.003 | 0.003 |
| 95% CI | [0.003, 0.004] | [0.002, 0.002] | [0.002, 0.003] | [0.002, 0.003] |
| Difference in initial level difference between treated and control ($\beta_{4}$) |  |  |  |  |
| Coefficient | 0.001 | -0.001 | -0.001 | -0.002 |
| 95% CI | [-0.027, 0.028] | [-0.022, 0.020] | [-0.042, 0.040] | [-0.032, 0.027] |
| Difference in pre-intervention trend between treated and control ($\beta_{5}$) |  |  |  |  |
| Coefficient | -0.000 | 0.000 | 0.000 | 0.000 |
| 95% CI | [-0.001, 0.001] | [-0.000, 0.000] | [-0.001, 0.001] | [-0.001, 0.001] |
| Immediate effect (level) of intervention ($\beta_{6}$) |  |  |  |  |
| Coefficient | -0.115*** | -0.017** | 0.023 | 0.001 |
| 95% CI | [-0.151,-0.080] | [-0.037, 0.002] | [-0.037, 0.083] | [-0.028, 0.030] |
| Effect on trend of intervention ($\beta_{7}$) |  |  |  |  |
| Coefficient | 0.005 | 0.000 | 0.002 | 0.000 |
| 95% CI | [0.004, 0.007] | [-0.001, 0.001] | [-0.000, 0.003] | [-0.001, 0.001] |

*** p<0.01; ** p<0.05; * p<0.1

Table S3: Estimated effect on monthly age-standardized hospitalization rates for stroke per 100,000 inhabitants (period 2007-2017) (One-tail significance test)

|  | 20-44 | 45-64 | 65+ | Entire population |
| --- | --- | --- | --- | --- |
|  | Log(rate) | Log(rate) | Log(rate) | Log(rate) |
| Initial control trend ($\beta_{1}$) |  |  |  |  |
| Coefficient | 0.003 | 0.001 | 0.000 | 0.000 |
| 95% CI | [0.003, 0.003] | [0.001, 0.001] |  | [0.000, 0.000] |
| Difference in initial level difference between treated and control ($\beta_{4}$) |  |  |  |  |
| Coefficient | -0.002 | -0.002 | 0.020 | -0.002 |
| 95% CI | [-0.018, 0.015] | [-0.020, 0.016] |  | [-0.007, 0.004] |
| Difference in pre-intervention trend between treated and control ($\beta_{5}$) |  |  |  |  |
| Coefficient | 0.000 | 0.000 | -0.000 | 0.000 |
| 95% CI | [-0.000, 0.000] | [-0.000, 0.001] |  | [-0.000, 0.000] |
| Immediate effect (level) of intervention ($\beta_{6}$) |  |  |  |  |
| Coefficient | 0.044 | 0.011 | -0.013 | -0.012*** |
| 95% CI | [0.024, 0.063] | [-0.014, 0.035] |  | [-0.020,-0.003] |
| Effect on trend of intervention ($\beta_{7}$) |  |  |  |  |
| Coefficient | -0.001* | -0.004*** | -0.003 | -0.002*** |
| 95% CI | [-0.001, 0.000] | [-0.005,-0.003] |  | [-0.003,-0.002] |

*** p<0.01; ** p<0.05; * p<0.1

Table S4: Estimated effect on monthly age-standardized hospitalization rates for the composite outcome per 100,000 inhabitants (period 2007-2017) (One-tail significance test)

|  | 20-44 | 45-64 | 65+ | Entire population |
| --- | --- | --- | --- | --- |
|  | Log(rate) | Log(rate) | Log(rate) | Log(rate) |
| Initial control trend ($\beta_{1}$) |  |  |  |  |
| Coefficient | 0.001 | -0.000*** | -0.000*** | -0.000*** |
| 95% CI | [0.001, 0.002] | [-0.000,-0.000] | [-0.000,-0.000] | [-0.000,-0.000] |
| Difference in initial level difference between treated and control ($\beta_{4}$) |  |  |  |  |
| Coefficient | -0.001 | 0.019 | 0.022 | 0.009 |
| 95% CI | [-0.025, 0.024] | [0.005, 0.033] | [0.006, 0.037] | [-0.001, 0.019] |
| Difference in pre-intervention trend between treated and control ($\beta_{5}$) |  |  |  |  |
| Coefficient | 0.000 | -0.000*** | -0.001*** | -0.000** |
| 95% CI | [-0.001, 0.001] | [-0.001,-0.000] | [-0.001,-0.000] | [-0.000, 0.000] |
| Immediate effect (level) of intervention ($\beta_{6}$) |  |  |  |  |
| Coefficient | -0.025* | -0.012 | -0.018* | -0.013 |
| 95% CI | [-0.058, 0.008] | [-0.030, 0.007] | [-0.040, 0.004] | [-0.034, 0.008] |
| Effect on trend of intervention ($\beta_{7}$) |  |  |  |  |
| Coefficient | 0.000 | 0.001 | -0.001*** | -0.001*** |
| 95% CI | [-0.000, 0.001] | [-0.000, 0.001] | [-0.002,-0.001] | [-0.001,-0.000] |

*** p<0.01; ** p<0.05; * p<0.1

Table S5: Estimated effect on monthly age-standardized deaths rates for ischemic heart disease per 100,000 inhabitants (period 2007-2017) (One-tail significance test)

|  | 20-44 | 45-64 | 65+ | Entire population |
| --- | --- | --- | --- | --- |
|  | Log(rate) | Log(rate) | Log(rate) | Log(rate) |
| Initial control trend ($\beta_{1}$) |  |  |  |  |
| Coefficient | -0.000 | -0.002*** | -0.004*** | -0.003*** |
| 95% CI | [-0.001, 0.001] | [-0.003,-0.002] | [-0.005,-0.003] | [-0.004,-0.002] |
| Difference in initial level difference between treated and control ($\beta_{4}$) |  |  |  |  |
| Coefficient | -0.005 | 0.003 | -0.002 | -0.001 |
| 95% CI | [-0.074, 0.064] | [-0.039, 0.045] | [-0.084, 0.080] | [-0.072, 0.069] |
| Difference in pre-intervention trend between treated and control ($\beta_{5}$) |  |  |  |  |
| Coefficient | 0.000 | -0.000 | 0.000 | 0.000 |
| 95% CI | [-0.001, 0.001] | [-0.001, 0.001] | [-0.002, 0.002] | [-0.001, 0.001] |
| Immediate effect (level) of intervention ($\beta_{6}$) |  |  |  |  |
| Coefficient | 0.076 | 0.081 | 0.138 | 0.086 |
| 95% CI | [-0.008, 0.160] | [0.018, 0.144] | [0.045, 0.231] | [0.021, 0.150] |
| Effect on trend of intervention ($\beta_{7}$) |  |  |  |  |
| Coefficient | -0.006*** | -0.005*** | -0.003** | -0.005*** |
| 95% CI | [-0.009,-0.003] | [-0.007,-0.004] | [-0.007,-0.000] | [-0.007,-0.003] |

*** p<0.01; ** p<0.05; * p<0.1

Table S6: Estimated effect on monthly age-standardized deaths rates for AMI per 100,000 inhabitants (period 2007-2017) (One-tail significance test)

|  | 20-44 | 45-64 | 65+ | Entire population |
| --- | --- | --- | --- | --- |
|  | Log(rate) | Log(rate) | Log(rate) | Log(rate) |
| Initial control trend ($\beta_{1}$) |  |  |  |  |
| Coefficient | -0.000 | -0.002*** | -0.003*** | -0.003*** |
| 95% CI | [-0.001, 0.000] | [-0.002,-0.002] | [-0.004,-0.002] | [-0.004,-0.002] |
| Difference in initial level difference between treated and control ($\beta_{4}$) |  |  |  |  |
| Coefficient | -0.011 | 0.004 | -0.002 | -0.001 |
| 95% CI | [-0.051, 0.029] | [-0.021, 0.029] | [-0.079, 0.074] | [-0.055, 0.053] |
| Difference in pre-intervention trend between treated and control ($\beta_{5}$) |  |  |  |  |
| Coefficient | 0.000 | -0.000 | 0.000 | -0.000 |
| 95% CI | [-0.001, 0.001] | [-0.001, 0.001] | [-0.002, 0.002] | [-0.001, 0.001] |
| Immediate effect (level) of intervention ($\beta_{6}$) |  |  |  |  |
| Coefficient | -0.015 | 0.056 | 0.098 | 0.027 |
| 95% CI | [-0.050, 0.021] | [0.021, 0.092] | [0.021, 0.174] | [-0.020, 0.073] |
| Effect on trend of intervention ($\beta_{7}$) |  |  |  |  |
| Coefficient | -0.005*** | -0.005*** | -0.004*** | -0.004*** |
| 95% CI | [-0.007,-0.004] | [-0.006,-0.004] | [-0.007,-0.002] | [-0.006,-0.003] |

*** p<0.01; ** p<0.05; * p<0.1

Table S7: Estimated effect on monthly age-standardized deaths rates for stroke per 100,000 inhabitants (period 2007-2017) (One-tail significance test)

|  | 20-44 | 45-64 | 65+ | Entire population |
| --- | --- | --- | --- | --- |
|  | Log(rate) | Log(rate) | Log(rate) | Log(rate) |
| Initial control trend ($\beta_{1}$) |  |  |  |  |
| Coefficient | -0.001*** | -0.003*** | -0.003*** | -0.003*** |
| 95% CI | [-0.001,-0.001] | [-0.003,-0.002] | [-0.004,-0.003] | [-0.004,-0.003] |
| Difference in initial level difference between treated and control ($\beta_{4}$) |  |  |  |  |
| Coefficient | 0.028 | 0.004 | 0.001 | 0.001 |
| 95% CI | [-0.017, 0.073] | [-0.019, 0.028] | [-0.034, 0.037] | [-0.033, 0.034] |
| Difference in pre-intervention trend between treated and control ($\beta_{5}$) |  |  |  |  |
| Coefficient | -0.001** | -0.000 | -0.000 | -0.000 |
| 95% CI | [-0.002, 0.000] | [-0.001, 0.000] | [-0.001, 0.001] | [-0.001, 0.001] |
| Immediate effect (level) of intervention ($\beta_{6}$) |  |  |  |  |
| Coefficient | -0.077** | 0.007 | 0.108 | 0.070 |
| 95% CI | [-0.166, 0.011] | [-0.028, 0.042] | [0.064, 0.152] | [0.035, 0.105] |
| Effect on trend of intervention ($\beta_{7}$) |  |  |  |  |
| Coefficient | -0.005*** | -0.009*** | -0.009*** | -0.008*** |
| 95% CI | [-0.009,-0.001] | [-0.010,-0.008] | [-0.011,-0.008] | [-0.009,-0.006] |

*** p<0.01; ** p<0.05; * p<0.1

Table S8: Estimated effect on monthly age-standardized deaths rates for the composite outcome per 100,000 inhabitants (period 2007-2017) (One-tail significance test)

|  | 20-44 | 45-64 | 65+ | Entire population |
| --- | --- | --- | --- | --- |
|  | Log(rate) | Log(rate) | Log(rate) | Log(rate) |
| Initial control trend ($\beta_{1}$) |  |  |  |  |
| Coefficient | -0.000** | -0.003*** | -0.004*** | -0.003*** |
| 95% CI | [-0.001,-0.000] | [-0.003,-0.002] | [-0.004,-0.003] | [-0.004,-0.002] |
| Difference in initial level difference between treated and control ($\beta_{4}$) |  |  |  |  |
| Coefficient | 0.003 | 0.002 | -0.001 | -0.001 |
| 95% CI | [-0.056, 0.062] | [-0.033, 0.037] | [-0.066, 0.064] | [-0.058, 0.057] |
| Difference in pre-intervention trend between treated and control ($\beta_{5}$) |  |  |  |  |
| Coefficient | -0.000 | -0.000 | 0.000 | 0.000 |
| 95% CI | [-0.001, 0.001] | [-0.001, 0.001] | [-0.001, 0.001] | [-0.001, 0.001] |
| Immediate effect (level) of intervention ($\beta_{6}$) |  |  |  |  |
| Coefficient | 0.024 | 0.055 | 0.120 | 0.075 |
| 95% CI | [-0.021, 0.069] | [0.004, 0.107] | [0.048, 0.191] | [0.026, 0.123] |
| Effect on trend of intervention ($\beta_{7}$) |  |  |  |  |
| Coefficient | -0.005*** | -0.007*** | -0.006*** | -0.006*** |
| 95% CI | [-0.006,-0.003] | [-0.008,-0.005] | [-0.009,-0.004] | [-0.008,-0.004] |

*** p<0.01; ** p<0.05; * p<0.1

**Results by sex (males)**

Table S9: Estimated effect on monthly male age-standardized hospitalization rates for ischemic heart disease per 100,000 inhabitants (period 2007-2017) (One-tail significance test)

|  | 20-44 | 45-64 | 65+ | Entire population |
| --- | --- | --- | --- | --- |
|  | Log(rate) | Log(rate) | Log(rate) | Log(rate) |
| Initial control trend ($\beta_{1}$) |  |  |  |  |
| Coefficient | 0.000 | -0.002*** | -0.001*** | -0.001*** |
| 95% CI | [0.000, 0.001] | [-0.002,-0.002] | [-0.001,-0.001] | [-0.002,-0.001] |
| Difference in initial level difference between treated and control ($\beta_{4}$) |  |  |  |  |
| Coefficient | -0.001 | 0.004 | 0.003 | 0.002 |
| 95% CI | [-0.026, 0.024] | [-0.020, 0.028] | [-0.012, 0.019] | [-0.013, 0.016] |
| Difference in pre-intervention trend between treated and control ($\beta_{5}$) |  |  |  |  |
| Coefficient | 0.000 | -0.000 | -0.000 | -0.000 |
| 95% CI | [-0.001, 0.001] | [-0.001, 0.000] | [-0.000, 0.000] | [-0.000, 0.000] |
| Immediate effect (level) of intervention ($\beta_{6}$) |  |  |  |  |
| Coefficient | -0.130*** | -0.032** | -0.013 | -0.009 |
| 95% CI | [-0.192,-0.068] | [-0.067, 0.003] | [-0.035, 0.010] | [-0.040, 0.023] |
| Effect on trend of intervention ($\beta_{7}$) |  |  |  |  |
| Coefficient | 0.002 | 0.002 | -0.000 | 0.001 |
| 95% CI | [0.001, 0.004] | [0.001, 0.003] | [-0.001, 0.001] | [0.000, 0.002] |

*** p<0.01; ** p<0.05; * p<0.1

Table S10: Estimated effect on monthly male age-standardized hospitalization rates for AMI per 100,000 inhabitants (period 2007-2017) (One-tail significance test)

|  | 20-44 | 45-64 | 65+ | Entire population |
| --- | --- | --- | --- | --- |
|  | Log(rate) | Log(rate) | Log(rate) | Log(rate) |
| Initial control trend ($\beta_{1}$) |  |  |  |  |
| Coefficient | 0.004 | 0.002 | 0.003 | 0.002 |
| 95% CI | [0.003, 0.005] | [0.001, 0.002] | [0.002, 0.003] | [0.002, 0.003] |
| Difference in initial level difference between treated and control ($\beta_{4}$) |  |  |  |  |
| Coefficient | -0.002 | -0.001 | -0.005 | -0.002 |
| 95% CI | [-0.051, 0.047] | [-0.024, 0.023] | [-0.037, 0.028] | [-0.026, 0.022] |
| Difference in pre-intervention trend between treated and control ($\beta_{5}$) |  |  |  |  |
| Coefficient | 0.000 | 0.000 | 0.000 | 0.000 |
| 95% CI | [-0.001, 0.001] | [-0.000, 0.000] | [-0.001, 0.001] | [-0.000, 0.000] |
| Immediate effect (level) of intervention ($\beta_{6}$) |  |  |  |  |
| Coefficient | -0.160*** | 0.002 | -0.022 | -0.006 |
| 95% CI | [-0.253,-0.067] | [-0.024, 0.027] | [-0.075, 0.032] | [-0.037, 0.024] |
| Effect on trend of intervention ($\beta_{7}$) |  |  |  |  |
| Coefficient | 0.008 | -0.000 | 0.000 | 0.001 |
| 95% CI | [0.005, 0.011] | [-0.001, 0.001] | [-0.001, 0.002] | [-0.000, 0.002] |

*** p<0.01; ** p<0.05; * p<0.1

Table S11: Estimated effect on monthly male age-standardized hospitalization rates for stroke per 100,000 inhabitants (period 2007-2017) (One-tail significance test)

|  | 20-44 | 45-64 | 65+ | Entire population |
| --- | --- | --- | --- | --- |
|  | Log(rate) | Log(rate) | Log(rate) | Log(rate) |
| Initial control trend ($\beta_{1}$) |  |  |  |  |
| Coefficient | 0.004 | 0.001 | 0.000 | 0.000 |
| 95% CI | [0.004, 0.005] | [0.001, 0.001] | [-0.000, 0.000] | [0.000, 0.000] |
| Difference in initial level difference between treated and control ($\beta_{4}$) |  |  |  |  |
| Coefficient | -0.005 | 0.000 | 0.011 | -0.003 |
| 95% CI | [-0.043, 0.032] | [-0.018, 0.019] | [-0.002, 0.025] | [-0.014, 0.008] |
| Difference in pre-intervention trend between treated and control ($\beta_{5}$) |  |  |  |  |
| Coefficient | 0.000 | 0.000 | -0.000 | 0.000 |
| 95% CI | [-0.001, 0.001] | [-0.000, 0.000] | [-0.000, 0.000] | [-0.000, 0.000] |
| Immediate effect (level) of intervention ($\beta_{6}$) |  |  |  |  |
| Coefficient | -0.005 | -0.001 | 0.007 | -0.001 |
| 95% CI | [-0.051, 0.041] | [-0.035, 0.034] | [-0.015, 0.029] | [-0.024, 0.021] |
| Effect on trend of intervention ($\beta_{7}$) |  |  |  |  |
| Coefficient | -0.000 | -0.003*** | -0.003*** | -0.003*** |
| 95% CI | [-0.002, 0.002] | [-0.005,-0.002] | [-0.004,-0.002] | [-0.003,-0.002] |

*** p<0.01; ** p<0.05; * p<0.1

Table S12: Estimated effect on monthly male age-standardized hospitalization rates for the composite outcome per 100,000 inhabitants (period 2007-2017) (One-tail significance test)

|  | 20-44 | 45-64 | 65+ | Entire population |
| --- | --- | --- | --- | --- |
|  | Log(rate) | Log(rate) | Log(rate) | Log(rate) |
| Initial control trend ($\beta_{1}$) |  |  |  |  |
| Coefficient | 0.002 | -0.000*** | -0.000** | -0.001*** |
| 95% CI | [0.002, 0.002] | [-0.001,-0.000] | [-0.000,-0.000] | [-0.001,-0.000] |
| Difference in initial level difference between treated and control ($\beta_{4}$) |  |  |  |  |
| Coefficient | -0.002 | 0.019 | 0.018 | 0.001 |
| 95% CI | [-0.028, 0.024] | [0.003, 0.034] | [0.008, 0.028] | [-0.008, 0.010] |
| Difference in pre-intervention trend between treated and control ($\beta_{5}$) |  |  |  |  |
| Coefficient | 0.000 | -0.000*** | -0.000*** | -0.000 |
| 95% CI | [-0.001, 0.001] | [-0.001,-0.000] | [-0.001,-0.000] | [-0.000, 0.000] |
| Immediate effect (level) of intervention ($\beta_{6}$) |  |  |  |  |
| Coefficient | -0.059*** | -0.023** | -0.003 | -0.010 |
| 95% CI | [-0.098,-0.021] | [-0.048, 0.002] | [-0.022, 0.015] | [-0.034, 0.014] |
| Effect on trend of intervention ($\beta_{7}$) |  |  |  |  |
| Coefficient | 0.002 | 0.001 | -0.001*** | -0.000 |
| 95% CI | [0.001, 0.003] | [0.000, 0.002] | [-0.002,-0.001] | [-0.001, 0.001] |

*** p<0.01; ** p<0.05; * p<0.1

Table S13: Estimated effect on monthly male age-standardized deaths rates for ischemic heart disease per 100,000 inhabitants (period 2007-2017) (One-tail significance test)

|  | 20-44 | 45-64 | 65+ | Entire population |
| --- | --- | --- | --- | --- |
|  | Log(rate) | Log(rate) | Log(rate) | Log(rate) |
| Initial control trend ($\beta_{1}$) |  |  |  |  |
| Coefficient | -0.001*** | -0.002*** | -0.003*** | -0.003*** |
| 95% CI | [-0.002,-0.001] | [-0.003,-0.001] | [-0.005,-0.002] | [-0.004,-0.002] |
| Difference in initial level difference between treated and control ($\beta_{4}$) |  |  |  |  |
| Coefficient | 0.001 | 0.011 | -0.000 | -0.001 |
| 95% CI | [-0.040, 0.042] | [-0.024, 0.046] | [-0.086, 0.085] | [-0.073, 0.071] |
| Difference in pre-intervention trend between treated and control ($\beta_{5}$) |  |  |  |  |
| Coefficient | -0.000 | -0.000 | -0.000 | -0.000 |
| 95% CI | [-0.001, 0.001] | [-0.001, 0.001] | [-0.002, 0.002] | [-0.002, 0.002] |
| Immediate effect (level) of intervention ($\beta_{6}$) |  |  |  |  |
| Coefficient | 0.122 | 0.073 | 0.029 | 0.039 |
| 95% CI | [0.048, 0.197] | [0.022, 0.123] | [-0.047, 0.105] | [-0.019, 0.097] |
| Effect on trend of intervention ($\beta_{7}$) |  |  |  |  |
| Coefficient | -0.007*** | -0.004*** | -0.003*** | -0.006*** |
| 95% CI | [-0.009,-0.005] | [-0.005,-0.003] | [-0.006,-0.001] | [-0.008,-0.004] |

*** p<0.01; ** p<0.05; * p<0.1

Table S14: Estimated effect on monthly male age-standardized deaths rates for AMI per 100,000 inhabitants (period 2007-2017) (One-tail significance test)

|  | 20-44 | 45-64 | 65+ | Entire population |
| --- | --- | --- | --- | --- |
|  | Log(rate) | Log(rate) | Log(rate) | Log(rate) |
| Initial control trend ($\beta_{1}$) |  |  |  |  |
| Coefficient | -0.001*** | -0.002*** | -0.003*** | -0.003*** |
| 95% CI | [-0.002,-0.001] | [-0.003,-0.001] | [-0.004,-0.002] | [-0.004,-0.002] |
| Difference in initial level difference between treated and control ($\beta_{4}$) |  |  |  |  |
| Coefficient | 0.000 | 0.001 | -0.001 | -0.001 |
| 95% CI | [-0.060, 0.060] | [-0.031, 0.033] | [-0.095, 0.094] | [-0.076, 0.075] |
| Difference in pre-intervention trend between treated and control ($\beta_{5}$) |  |  |  |  |
| Coefficient | -0.000 | -0.000 | 0.000 | -0.000 |
| 95% CI | [-0.001, 0.001] | [-0.001, 0.001] | [-0.002, 0.002] | [-0.002, 0.002] |
| Immediate effect (level) of intervention ($\beta_{6}$) |  |  |  |  |
| Coefficient | 0.076 | 0.046 | 0.036 | 0.008 |
| 95% CI | [0.012, 0.139] | [-0.002, 0.094] | [-0.047, 0.119] | [-0.043, 0.059] |
| Effect on trend of intervention ($\beta_{7}$) |  |  |  |  |
| Coefficient | -0.007*** | -0.005*** | -0.005*** | -0.006*** |
| 95% CI | [-0.009,-0.004] | [-0.007,-0.003] | [-0.008,-0.003] | [-0.008,-0.004] |

*** p<0.01; ** p<0.05; * p<0.1

Table S15: Estimated effect on monthly male age-standardized deaths rates for stroke per 100,000 inhabitants (period 2007-2017) (One-tail significance test)

|  | 20-44 | 45-64 | 65+ | Entire population |
| --- | --- | --- | --- | --- |
|  | Log(rate) | Log(rate) | Log(rate) | Log(rate) |
| Initial control trend ($\beta_{1}$) |  |  |  |  |
| Coefficient | -0.000 | -0.003*** | -0.003*** | -0.003*** |
| 95% CI | [-0.000, 0.000] | [-0.003,-0.002] | [-0.003,-0.003] | [-0.003,-0.003] |
| Difference in initial level difference between treated and control ($\beta_{4}$) |  |  |  |  |
| Coefficient | 0.052 | 0.005 | 0.002 | 0.000 |
| 95% CI | [0.017, 0.087] | [-0.026, 0.035] | [-0.027, 0.031] | [-0.027, 0.028] |
| Difference in pre-intervention trend between treated and control ($\beta_{5}$) |  |  |  |  |
| Coefficient | -0.001*** | -0.000 | -0.000 | -0.000 |
| 95% CI | [-0.002,-0.000] | [-0.001, 0.001] | [-0.001, 0.000] | [-0.001, 0.001] |
| Immediate effect (level) of intervention ($\beta_{6}$) |  |  |  |  |
| Coefficient | -0.010 | -0.048* | 0.061 | 0.003 |
| 95% CI | [-0.135, 0.115] | [-0.113, 0.017] | [0.022, 0.101] | [-0.027, 0.032] |
| Effect on trend of intervention ($\beta_{7}$) |  |  |  |  |
| Coefficient | -0.004 | -0.007*** | -0.009*** | -0.008*** |
| 95% CI | [-0.011, 0.004] | [-0.008,-0.005] | [-0.010,-0.008] | [-0.009,-0.007] |

*** p<0.01; ** p<0.05; * p<0.1

Table S16: Estimated effect on monthly male age-standardized deaths rates for the composite outcome per 100,000 inhabitants (period 2007-2017) (One-tail significance test)

|  | 20-44 | 45-64 | 65+ | Entire population |
| --- | --- | --- | --- | --- |
|  | Log(rate) | Log(rate) | Log(rate) | Log(rate) |
| Initial control trend ($\beta_{1}$) |  |  |  |  |
| Coefficient | -0.001*** | -0.002*** | -0.003*** | -0.003*** |
| 95% CI | [-0.001,-0.000] | [-0.003,-0.002] | [-0.004,-0.002] | [-0.004,-0.002] |
| Difference in initial level difference between treated and control ($\beta_{4}$) |  |  |  |  |
| Coefficient | 0.017 | 0.003 | 0.000 | -0.000 |
| 95% CI | [-0.019, 0.054] | [-0.030, 0.035] | [-0.059, 0.059] | [-0.051, 0.050] |
| Difference in pre-intervention trend between treated and control ($\beta_{5}$) |  |  |  |  |
| Coefficient | -0.001* | -0.000 | -0.000 | -0.000 |
| 95% CI | [-0.001, 0.000] | [-0.001, 0.001] | [-0.001, 0.001] | [-0.001, 0.001] |
| Immediate effect (level) of intervention ($\beta_{6}$) |  |  |  |  |
| Coefficient | 0.104 | 0.032 | 0.073 | 0.036 |
| 95% CI | [0.055, 0.153] | [-0.020, 0.084] | [0.016, 0.129] | [-0.005, 0.077] |
| Effect on trend of intervention ($\beta_{7}$) |  |  |  |  |
| Coefficient | -0.006*** | -0.005*** | -0.006*** | -0.006*** |
| 95% CI | [-0.008,-0.004] | [-0.006,-0.004] | [-0.008,-0.005] | [-0.008,-0.005] |

*** p<0.01; ** p<0.05; * p<0.1

**Results by sex (females)**

Table S17: Estimated effect on monthly female age-standardized hospitalization rates for ischemic heart disease per 100,000 inhabitants (period 2007-2017) (One-tail significance test)

|  | 20-44 | 45-64 | 65+ | Entire population |
| --- | --- | --- | --- | --- |
|  | Log(rate) | Log(rate) | Log(rate) | Log(rate) |
| Initial control trend ($\beta_{1}$) |  |  |  |  |
| Coefficient | -0.004*** | -0.002*** | -0.002*** | -0.002*** |
| 95% CI | [-0.005,-0.003] | [-0.003,-0.002] | [-0.002,-0.001] | [-0.003,-0.002] |
| Difference in initial level difference between treated and control ($\beta_{4}$) |  |  |  |  |
| Coefficient | 0.008 | -0.000 | -0.000 | -0.000 |
| 95% CI | [-0.036, 0.051] | [-0.016, 0.016] | [-0.055, 0.055] | [-0.033, 0.032] |
| Difference in pre-intervention trend between treated and control ($\beta_{5}$) |  |  |  |  |
| Coefficient | -0.000 | -0.000 | -0.000 | -0.000 |
| 95% CI | [-0.002, 0.002] | [-0.000, 0.000] | [-0.001, 0.001] | [-0.001, 0.001] |
| Immediate effect (level) of intervention ($\beta_{6}$) |  |  |  |  |
| Coefficient | -0.055 | -0.008 | -0.019 | -0.007 |
| 95% CI | [-0.189, 0.079] | [-0.034, 0.019] | [-0.080, 0.041] | [-0.046, 0.031] |
| Effect on trend of intervention ($\beta_{7}$) |  |  |  |  |
| Coefficient | -0.003* | 0.001 | 0.002 | 0.002 |
| 95% CI | [-0.008, 0.001] | [0.001, 0.002] | [0.000, 0.004] | [0.000, 0.003] |

*** p<0.01; ** p<0.05; * p<0.1

Table S18: Estimated effect on monthly female age-standardized hospitalization rates for AMI per 100,000 inhabitants (period 2007-2017) (One-tail significance test)

|  | 20-44 | 45-64 | 65+ | Entire population |
| --- | --- | --- | --- | --- |
|  | Log(rate) | Log(rate) | Log(rate) | Log(rate) |
| Initial control trend ($\beta_{1}$) |  |  |  |  |
| Coefficient | 0.003 | 0.004 | 0.002 | 0.003 |
| 95% CI | [0.001, 0.004] | [0.003, 0.005] | [0.001, 0.003] | [0.002, 0.004] |
| Difference in initial level difference between treated and control ($\beta_{4}$) |  |  |  |  |
| Coefficient | 0.004 | -0.003 | -0.001 | -0.004 |
| 95% CI | [-0.140, 0.148] | [-0.080, 0.074] | [-0.067, 0.066] | [-0.069, 0.062] |
| Difference in pre-intervention trend between treated and control ($\beta_{5}$) |  |  |  |  |
| Coefficient | -0.000 | 0.000 | 0.000 | 0.000 |
| 95% CI | [-0.003, 0.003] | [-0.001, 0.002] | [-0.001, 0.001] | [-0.001, 0.001] |
| Immediate effect (level) of intervention ($\beta_{6}$) |  |  |  |  |
| Coefficient | -0.058 | -0.051** | 0.031 | -0.020 |
| 95% CI | [-0.192, 0.076] | [-0.106, 0.005] | [-0.035, 0.097] | [-0.063, 0.023] |
| Effect on trend of intervention ($\beta_{7}$) |  |  |  |  |
| Coefficient | 0.004 | 0.006 | 0.002 | 0.001 |
| 95% CI | [-0.000, 0.009] | [0.005, 0.008] | [-0.000, 0.004] | [-0.001, 0.003] |

*** p<0.01; ** p<0.05; * p<0.1

Table S19: Estimated effect on monthly female age-standardized hospitalization rates for stroke per 100,000 inhabitants (period 2007-2017) (One-tail significance test)

|  | 20-44 | 45-64 | 65+ | Entire population |
| --- | --- | --- | --- | --- |
|  | Log(rate) | Log(rate) | Log(rate) | Log(rate) |
| Initial control trend ($\beta_{1}$) |  |  |  |  |
| Coefficient | 0.002 | 0.001 | -0.001*** | 0.000 |
| 95% CI | [0.001, 0.002] | [0.001, 0.002] | [-0.001,-0.000] | [-0.000, 0.000] |
| Difference in initial level difference between treated and control ($\beta_{4}$) |  |  |  |  |
| Coefficient | -0.004 | 0.000 | 0.002 | -0.001 |
| 95% CI | [-0.061, 0.052] | [-0.043, 0.043] | [-0.022, 0.026] | [-0.015, 0.014] |
| Difference in pre-intervention trend between treated and control ($\beta_{5}$) |  |  |  |  |
| Coefficient | 0.000 | 0.000 | -0.000 | 0.000 |
| 95% CI | [-0.001, 0.001] | [-0.001, 0.001] | [-0.000, 0.000] | [-0.000, 0.000] |
| Immediate effect (level) of intervention ($\beta_{6}$) |  |  |  |  |
| Coefficient | 0.096 | 0.034 | 0.000 | -0.009 |
| 95% CI | [0.030, 0.162] | [-0.001, 0.069] | [-0.030, 0.030] | [-0.033, 0.014] |
| Effect on trend of intervention ($\beta_{7}$) |  |  |  |  |
| Coefficient | -0.001 | -0.007*** | -0.004*** | -0.002*** |
| 95% CI | [-0.003, 0.001] | [-0.009,-0.006] | [-0.005,-0.003] | [-0.003,-0.001] |

*** p<0.01; ** p<0.05; * p<0.1

Table S20: Estimated effect on monthly female age-standardized hospitalization rates for the composite outcome per 100,000 inhabitants (period 2007-2017) (One-tail significance test)

|  | 20-44 | 45-64 | 65+ | Entire population |
| --- | --- | --- | --- | --- |
|  | Log(rate) | Log(rate) | Log(rate) | Log(rate) |
| Initial control trend ($\beta_{1}$) |  |  |  |  |
| Coefficient | 0.000 | -0.000*** | -0.001*** | -0.001*** |
| 95% CI | [-0.001, 0.001] | [-0.001,-0.000] | [-0.001,-0.001] | [-0.001,-0.001] |
| Difference in initial level difference between treated and control ($\beta_{4}$) |  |  |  |  |
| Coefficient | 0.005 | 0.007 | 0.001 | 0.002 |
| 95% CI | [-0.043, 0.054] | [-0.011, 0.025] | [-0.033, 0.034] | [-0.012, 0.016] |
| Difference in pre-intervention trend between treated and control ($\beta_{5}$) |  |  |  |  |
| Coefficient | -0.000 | -0.000 | -0.000 | -0.000 |
| 95% CI | [-0.001, 0.001] | [-0.001, 0.000] | [-0.001, 0.001] | [-0.000, 0.000] |
| Immediate effect (level) of intervention ($\beta_{6}$) |  |  |  |  |
| Coefficient | 0.059 | -0.020** | -0.016 | -0.005 |
| 95% CI | [-0.015, 0.133] | [-0.038,-0.001] | [-0.053, 0.022] | [-0.031, 0.020] |
| Effect on trend of intervention ($\beta_{7}$) |  |  |  |  |
| Coefficient | 0.001 | -0.002*** | -0.002*** | -0.002*** |
| 95% CI | [-0.002, 0.003] | [-0.003,-0.001] | [-0.003,-0.001] | [-0.002,-0.001] |

*** p<0.01; ** p<0.05; * p<0.1

Table S21: Estimated effect on monthly female age-standardized deaths rates for ischemic heart disease per 100,000 inhabitants (period 2007-2017) (One-tail significance test)

|  | 20-44 | 45-64 | 65+ | Entire population |
| --- | --- | --- | --- | --- |
|  | Log(rate) | Log(rate) | Log(rate) | Log(rate) |
| Initial control trend ($\beta_{1}$) |  |  |  |  |
| Coefficient | 0.005 | -0.003*** | -0.005*** | -0.004*** |
| 95% CI | [0.002, 0.008] | [-0.005,-0.002] | [-0.006,-0.003] | [-0.005,-0.003] |
| Difference in initial level difference between treated and control ($\beta_{4}$) |  |  |  |  |
| Coefficient | -0.061 | 0.002 | -0.002 | -0.003 |
| 95% CI | [-0.303, 0.181] | [-0.062, 0.066] | [-0.086, 0.081] | [-0.078, 0.072] |
| Difference in pre-intervention trend between treated and control ($\beta_{5}$) |  |  |  |  |
| Coefficient | 0.001 | -0.000 | 0.000 | 0.000 |
| 95% CI | [-0.003, 0.006] | [-0.002, 0.002] | [-0.002, 0.002] | [-0.002, 0.002] |
| Immediate effect (level) of intervention ($\beta_{6}$) |  |  |  |  |
| Coefficient | -0.321*** | 0.108 | 0.106 | 0.072 |
| 95% CI | [-0.542,-0.100] | [0.008, 0.209] | [0.006, 0.205] | [-0.003, 0.147] |
| Effect on trend of intervention ($\beta_{7}$) |  |  |  |  |
| Coefficient | 0.001 | -0.006*** | 0.005 | -0.001 |
| 95% CI | [-0.007, 0.009] | [-0.008,-0.003] | [0.001, 0.009] | [-0.003, 0.002] |

*** p<0.01; ** p<0.05; * p<0.1

Table S22: Estimated effect on monthly female age-standardized deaths rates for AMI per 100,000 inhabitants (period 2007-2017) (One-tail significance test)

|  | 20-44 | 45-64 | 65+ | Entire population |
| --- | --- | --- | --- | --- |
|  | Log(rate) | Log(rate) | Log(rate) | Log(rate) |
| Initial control trend ($\beta_{1}$) |  |  |  |  |
| Coefficient | 0.004 | -0.003*** | -0.004*** | -0.003*** |
| 95% CI | [0.001, 0.008] | [-0.004,-0.002] | [-0.005,-0.002] | [-0.004,-0.002] |
| Difference in initial level difference between treated and control ($\beta_{4}$) |  |  |  |  |
| Coefficient | -0.099 | 0.001 | -0.004 | -0.003 |
| 95% CI | [-0.384, 0.186] | [-0.057, 0.059] | [-0.091, 0.082] | [-0.082, 0.076] |
| Difference in pre-intervention trend between treated and control ($\beta_{5}$) |  |  |  |  |
| Coefficient | 0.002 | -0.000 | 0.000 | 0.000 |
| 95% CI | [-0.003, 0.008] | [-0.002, 0.002] | [-0.002, 0.002] | [-0.002, 0.002] |
| Immediate effect (level) of intervention ($\beta_{6}$) |  |  |  |  |
| Coefficient | -0.432*** | 0.137 | 0.086 | 0.038 |
| 95% CI | [-0.689,-0.176] | [0.046, 0.227] | [0.013, 0.160] | [-0.027, 0.103] |
| Effect on trend of intervention ($\beta_{7}$) |  |  |  |  |
| Coefficient | 0.003 | -0.006*** | 0.003 | 0.002 |
| 95% CI | [-0.006, 0.012] | [-0.009,-0.004] | [0.001, 0.006] | [-0.001, 0.005] |

*** p<0.01; ** p<0.05; * p<0.1

Table S23: Estimated effect on monthly female age-standardized deaths rates for stroke per 100,000 inhabitants (period 2007-2017) (One-tail significance test)

|  | 20-44 | 45-64 | 65+ | Entire population |
| --- | --- | --- | --- | --- |
|  | Log(rate) | Log(rate) | Log(rate) | Log(rate) |
| Initial control trend ($\beta_{1}$) |  |  |  |  |
| Coefficient | -0.002*** | -0.003*** | -0.003*** | -0.003*** |
| 95% CI | [-0.003,-0.000] | [-0.004,-0.002] | [-0.004,-0.003] | [-0.004,-0.003] |
| Difference in initial level difference between treated and control ($\beta_{4}$) |  |  |  |  |
| Coefficient | 0.022 | 0.006 | 0.000 | -0.000 |
| 95% CI | [-0.097, 0.140] | [-0.057, 0.068] | [-0.066, 0.066] | [-0.066, 0.066] |
| Difference in pre-intervention trend between treated and control ($\beta_{5}$) |  |  |  |  |
| Coefficient | -0.001 | -0.000 | -0.000 | -0.000 |
| 95% CI | [-0.003, 0.002] | [-0.002, 0.001] | [-0.001, 0.001] | [-0.001, 0.001] |
| Immediate effect (level) of intervention ($\beta_{6}$) |  |  |  |  |
| Coefficient | -0.257*** | 0.079 | 0.070 | 0.051 |
| 95% CI | [-0.409,-0.105] | [-0.011, 0.169] | [0.008, 0.133] | [-0.000, 0.102] |
| Effect on trend of intervention ($\beta_{7}$) |  |  |  |  |
| Coefficient | -0.007*** | -0.011*** | -0.003*** | -0.005*** |
| 95% CI | [-0.012,-0.003] | [-0.014,-0.008] | [-0.005,-0.001] | [-0.007,-0.003] |

*** p<0.01; ** p<0.05; * p<0.1

Table S24: Estimated effect on monthly female age-standardized deaths rates for the composite outcome per 100,000 inhabitants (period 2007-2017) (One-tail significance test)

|  | 20-44 | 45-64 | 65+ | Entire population |
| --- | --- | --- | --- | --- |
|  | Log(rate) | Log(rate) | Log(rate) | Log(rate) |
| Initial control trend ($\beta_{1}$) |  |  |  |  |
| Coefficient | -0.000 | -0.003*** | -0.004*** | -0.004*** |
| 95% CI | [-0.001, 0.001] | [-0.004,-0.002] | [-0.005,-0.003] | [-0.005,-0.003] |
| Difference in initial level difference between treated and control ($\beta_{4}$) |  |  |  |  |
| Coefficient | -0.042 | 0.001 | -0.001 | -0.002 |
| 95% CI | [-0.178, 0.095] | [-0.049, 0.051] | [-0.074, 0.072] | [-0.070, 0.066] |
| Difference in pre-intervention trend between treated and control ($\beta_{5}$) |  |  |  |  |
| Coefficient | 0.001 | -0.000 | 0.000 | 0.000 |
| 95% CI | [-0.002, 0.003] | [-0.001, 0.001] | [-0.001, 0.001] | [-0.001, 0.001] |
| Immediate effect (level) of intervention ($\beta_{6}$) |  |  |  |  |
| Coefficient | -0.219*** | 0.103 | 0.089 | 0.064 |
| 95% CI | [-0.339,-0.100] | [0.046, 0.160] | [0.004, 0.175] | [0.007, 0.121] |
| Effect on trend of intervention ($\beta_{7}$) |  |  |  |  |
| Coefficient | -0.005*** | -0.008*** | 0.002 | -0.003*** |
| 95% CI | [-0.009,-0.001] | [-0.010,-0.006] | [-0.001, 0.005] | [-0.005,-0.001] |

*** p<0.01; ** p<0.05; * p<0.1

**Main results with two-tails significance tests**

Table S25: Estimated effect on monthly age-standardized hospitalization rates for ischemic heart disease per 100,000 inhabitants (period 2007-2017) (Two-tails significance test)

|  | 20-44 | 45-64 | 65+ | Entire population |
| --- | --- | --- | --- | --- |
|  | Log(rate) | Log(rate) | Log(rate) | Log(rate) |
| Initial control trend ($\beta_{1}$) |  |  |  |  |
| Coefficient | -0.001*** | -0.002*** | -0.001*** | -0.002*** |
| 95% CI | [-0.00,-0.000] | [-0.002,-0.002] | [-0.001,-0.001] | [-0.002,-0.001] |
| Difference in initial level difference between treated and control ($\beta_{4}$) |  |  |  |  |
| Coefficient | 0.000 | 0.003 | -0.000 | 0.000 |
| 95% CI | [-0.013, 0.014] | [-0.006, 0.012] | [-0.024, 0.023] | [-0.015, 0.016] |
| Difference in pre-intervention trend between treated and control ($\beta_{5}$) |  |  |  |  |
| Coefficient | -0.000 | -0.000 | -0.000 | -0.000 |
| 95% CI | [-0.000, 0.000] | [-0.000, 0.000] | [-0.001, 0.001] | [-0.000, 0.000] |
| Immediate effect (level) of intervention ($\beta_{6}$) |  |  |  |  |
| Coefficient | -0.087*** | -0.015** | -0.040** | -0.001 |
| 95% CI | [-0.12,-0.054] | [-0.027,-0.002] | [-0.073,-0.007] | [-0.026, 0.024] |
| Effect on trend of intervention ($\beta_{7}$) |  |  |  |  |
| Coefficient | 0.001 | 0.003*** | 0.002*** | -0.000 |
| 95% CI | [-0.000, 0.002] | [0.003, 0.003] | [0.001, 0.003] | [-0.001, 0.000] |

*** p<0.01; ** p<0.05; * p<0.1

|  | 20-44 | 45-64 | 65+ | Entire population |
| --- | --- | --- | --- | --- |
|  | Log(rate) | Log(rate) | Log(rate) | Log(rate) |
| Initial control trend ($\beta_{1}$) |  |  |  |  |
| Coefficient | -0.000 | -0.002*** | -0.004*** | -0.003*** |
| 95% CI | [-0.000, 0.000] | [-0.003,-0.002] | [-0.005,-0.003] | [-0.004,-0.002] |
| Difference in initial level difference between treated and control ($\beta_{4}$) |  |  |  |  |
| Coefficient | -0.005 | 0.003 | -0.002 | -0.001 |
| 95% CI | [-0.038, 0.028] | [-0.030, 0.036] | [-0.074, 0.070] | [-0.062, 0.060] |
| Difference in pre-intervention trend between treated and control ($\beta_{5}$) |  |  |  |  |
| Coefficient | 0.000 | -0.000 | 0.000 | 0.000 |
| 95% CI | [-0.001, 0.001] | [-0.001, 0.001] | [-0.001, 0.002] | [-0.001, 0.001] |
| Immediate effect (level) of intervention ($\beta_{6}$) |  |  |  |  |
| Coefficient | 0.076*** | 0.081*** | 0.138*** | 0.086*** |
| 95% CI | [0.034, 0.119] | [0.031, 0.131] | [0.055, 0.222] | [0.028, 0.144] |
| Effect on trend of intervention ($\beta_{7}$) |  |  |  |  |
| Coefficient | -0.006*** | -0.005*** | -0.003** | -0.005*** |
| 95% CI | [-0.008-0.005] | [-0.006,-0.004] | [-0.006,-0.000] | [-0.007,-0.003] |

Table S26: Estimated effect on monthly age-standardized death rates for ischemic heart disease per 100,000 inhabitants (period 2007-2017) (Two-tails significance test)

*** p<0.01; ** p<0.05; * p<0.1

Table S27: Estimated effect on monthly age-standardized hospitalization rates for AMI per 100,000 inhabitants (period 2007-2017) (Two-tails significance test)

|  | 20-44 | 45-64 | 65+ | Entire population |
| --- | --- | --- | --- | --- |
|  | Log(rate) | Log(rate) | Log(rate) | Log(rate) |
| Initial control trend ($\beta_{1}$) |  |  |  |  |
| Coefficient | 0.004*** | 0.002*** | 0.003*** | 0.003*** |
| 95% CI | [0.003, 0.004] | [0.002, 0.002] | [0.002, 0.003] | [0.002, 0.003] |
| Difference in initial level difference between treated and control ($\beta_{4}$) |  |  |  |  |
| Coefficient | 0.001 | -0.001 | -0.001 | -0.002 |
| 95% CI | [-0.027, 0.028] | [-0.022, 0.020] | [-0.042, 0.040] | [-0.032, 0.027] |
| Difference in pre-intervention trend between treated and control ($\beta_{5}$) |  |  |  |  |
| Coefficient | -0.000 | 0.000 | 0.000 | 0.000 |
| 95% CI | [-0.001, 0.001] | [-0.000, 0.000] | [-0.001, 0.001] | [-0.001, 0.001] |
| Immediate effect (level) of intervention ($\beta_{6}$) |  |  |  |  |
| Coefficient | -0.115*** | -0.017* | 0.023 | 0.001 |
| 95% CI | [-0.151-0.080] | [-0.037, 0.002] | [-0.037, 0.083] | [-0.028, 0.030] |
| Effect on trend of intervention ($\beta_{7}$) |  |  |  |  |
| Coefficient | 0.005*** | 0.000 | 0.002* | 0.000 |
| 95% CI | [0.004, 0.007] | [-0.001, 0.001] | [-0.000, 0.003] | [-0.001, 0.001] |

*** p<0.01; ** p<0.05; * p<0.1

Table S28: Estimated effect on monthly age-standardized death rates for AMI per 100,000 inhabitants (period 2007-2017) (Two-tails significance test)

|  | 20-44 | 45-64 | 65+ | Entire population |
| --- | --- | --- | --- | --- |
|  | Log(rate) | Log(rate) | Log(rate) | Log(rate) |
| Initial control trend ($\beta_{1}$) |  |  |  |  |
| Coefficient | -0.000 | -0.002*** | -0.003*** | -0.003*** |
| 95% CI | [-0.001, 0.000] | [-0.002,-0.002] | [-0.004,-0.002] | [-0.004,-0.002] |
| Difference in initial level difference between treated and control ($\beta_{4}$) |  |  |  |  |
| Coefficient | -0.011 | 0.004 | -0.002 | -0.001 |
| 95% CI | [-0.051, 0.029] | [-0.021, 0.029] | [-0.079, 0.074] | [-0.055, 0.053] |
| Difference in pre-intervention trend between treated and control ($\beta_{5}$) |  |  |  |  |
| Coefficient | 0.000 | -0.000 | 0.000 | -0.000 |
| 95% CI | [-0.001, 0.001] | [-0.001, 0.001] | [-0.002, 0.002] | [-0.001, 0.001] |
| Immediate effect (level) of intervention ($\beta_{6}$) |  |  |  |  |
| Coefficient | -0.015 | 0.056*** | 0.098** | 0.027 |
| 95% CI | [-0.050, 0.021] | [0.021, 0.092] | [0.021, 0.174] | [-0.020, 0.073] |
| Effect on trend of intervention ($\beta_{7}$) |  |  |  |  |
| Coefficient | -0.005*** | -0.005*** | -0.004*** | -0.004*** |
| 95% CI | [-0.007-0.004] | [-0.006,-0.004] | [-0.007,-0.002] | [-0.006,-0.003] |

*** p<0.01; ** p<0.05; * p<0.1

Table S29: Estimated effect on monthly age-standardized hospitalization rates for stroke per 100,000 inhabitants (period 2007-2017) (Two-tails significance test)

|  | 20-44 | 45-64 | 65+ | Entire population |
| --- | --- | --- | --- | --- |
|  | Log(rate) | Log(rate) | Log(rate) | Log(rate) |
| Initial control trend ($\beta_{1}$) |  |  |  |  |
| Coefficient | 0.003*** | 0.001*** | 0.000 | 0.000*** |
| 95% CI | [0.003, 0.003] | [0.001, 0.001] |  | [0.000, 0.000] |
| Difference in initial level difference between treated and control ($\beta_{4}$) |  |  |  |  |
| Coefficient | -0.002 | -0.002 | 0.020 | -0.002 |
| 95% CI | [-0.018, 0.015] | [-0.020, 0.016] |  | [-0.007, 0.004] |
| Difference in pre-intervention trend between treated and control ($\beta_{5}$) |  |  |  |  |
| Coefficient | 0.000 | 0.000 | -0.000 | 0.000 |
| 95% CI | [-0.000, 0.000] | [-0.000, 0.001] |  | [-0.000, 0.000] |
| Immediate effect (level) of intervention ($\beta_{6}$) |  |  |  |  |
| Coefficient | 0.044*** | 0.011 | -0.013 | -0.012*** |
| 95% CI | [0.024, 0.063] | [-0.014, 0.035] |  | [-0.020,-0.003] |
| Effect on trend of intervention ($\beta_{7}$) |  |  |  |  |
| Coefficient | -0.001 | -0.004*** | -0.003 | -0.002*** |
| 95% CI | [-0.001, 0.000] | [-0.005,-0.003] |  | [-0.003,-0.002] |

*** p<0.01; ** p<0.05; * p<0.1

Table S30: Estimated effect on monthly age-standardized death rates for stroke per 100,000 inhabitants (period 2007-2017) (Two-tails significance test)

|  | 20-44 | 45-64 | 65+ | Entire population |
| --- | --- | --- | --- | --- |
|  | Log(rate) | Log(rate) | Log(rate) | Log(rate) |
| Initial control trend ($\beta_{1}$) |  |  |  |  |
| Coefficient | -0.001*** | -0.003*** | -0.003*** | -0.003*** |
| 95% CI | [-0.001-0.001] | [-0.003,-0.002] | [-0.004,-0.003] | [-0.004,-0.003] |
| Difference in initial level difference between treated and control ($\beta_{4}$) |  |  |  |  |
| Coefficient | 0.028 | 0.004 | 0.001 | 0.001 |
| 95% CI | [-0.017, 0.073] | [-0.019, 0.028] | [-0.034, 0.037] | [-0.033, 0.034] |
| Difference in pre-intervention trend between treated and control ($\beta_{5}$) |  |  |  |  |
| Coefficient | -0.001* | -0.000 | -0.000 | -0.000 |
| 95% CI | [-0.002, 0.000] | [-0.001, 0.000] | [-0.001, 0.001] | [-0.001, 0.001] |
| Immediate effect (level) of intervention ($\beta_{6}$) |  |  |  |  |
| Coefficient | -0.077* | 0.007 | 0.108*** | 0.070*** |
| 95% CI | [-0.166, 0.011] | [-0.028, 0.042] | [0.064, 0.152] | [0.035, 0.105] |
| Effect on trend of intervention ($\beta_{7}$) |  |  |  |  |
| Coefficient | -0.005** | -0.009*** | -0.009*** | -0.008*** |
| 95% CI | [-0.009-0.001] | [-0.010,-0.008] | [-0.011,-0.008] | [-0.009,-0.006] |

*** p<0.01; ** p<0.05; * p<0.1

Table S31: Estimated effect on monthly age-standardized hospitalization rates for the composite outcome per 100,000 inhabitants (period 2007-2017) (Two-tails significance test)

|  | 20-44 | 45-64 | 65+ | Entire population |
| --- | --- | --- | --- | --- |
|  | Log(rate) | Log(rate) | Log(rate) | Log(rate) |
| Initial control trend ($\beta_{1}$) |  |  |  |  |
| Coefficient | 0.001*** | -0.000*** | -0.000*** | -0.000*** |
| 95% CI | [0.001, 0.002] | [-0.000,-0.000] | [-0.000,-0.000] | [-0.000,-0.000] |
| Difference in initial level difference between treated and control ($\beta_{4}$) |  |  |  |  |
| Coefficient | -0.001 | 0.019*** | 0.022*** | 0.009* |
| 95% CI | [-0.025, 0.024] | [0.005, 0.033] | [0.006, 0.037] | [-0.001, 0.019] |
| Difference in pre-intervention trend between treated and control ($\beta_{5}$) |  |  |  |  |
| Coefficient | 0.000 | -0.000*** | -0.001*** | -0.000* |
| 95% CI | [-0.001, 0.001] | [-0.001,-0.000] | [-0.001,-0.000] | [-0.000, 0.000] |
| Immediate effect (level) of intervention ($\beta_{6}$) |  |  |  |  |
| Coefficient | -0.025 | -0.012 | -0.018 | -0.013 |
| 95% CI | [-0.058, 0.008] | [-0.030, 0.007] | [-0.040, 0.004] | [-0.034, 0.008] |
| Effect on trend of intervention ($\beta_{7}$) |  |  |  |  |
| Coefficient | 0.000 | 0.001 | -0.001*** | -0.001** |
| 95% CI | [-0.000, 0.001] | [-0.000, 0.001] | [-0.002,-0.001] | [-0.001,-0.000] |

*** p<0.01; ** p<0.05; * p<0.1

Table S32: Estimated effect on monthly age-standardized death rates for the composite outcome per 100,000 inhabitants (period 2007-2017) (Two-tails significance test)

|  | 20-44 | 45-64 | 65+ | Entire population |
| --- | --- | --- | --- | --- |
|  | Log(rate) | Log(rate) | Log(rate) | Log(rate) |
| Initial control trend ($\beta_{1}$) |  |  |  |  |
| Coefficient | -0.000** | -0.003*** | -0.004*** | -0.003*** |
| 95% CI | [-0.001,-0.000] | [-0.003,-0.002] | [-0.004,-0.003] | [-0.004,-0.002] |
| Difference in initial level difference between treated and control ($\beta_{4}$) |  |  |  |  |
| Coefficient | 0.003 | 0.002 | -0.001 | -0.001 |
| 95% CI | [-0.056, 0.062] | [-0.033, 0.037] | [-0.066, 0.064] | [-0.058, 0.057] |
| Difference in pre-intervention trend between treated and control ($\beta_{5}$) |  |  |  |  |
| Coefficient | -0.000 | -0.000 | 0.000 | 0.000 |
| 95% CI | [-0.001, 0.001] | [-0.001, 0.001] | [-0.001, 0.001] | [-0.001, 0.001] |
| Immediate effect (level) of intervention ($\beta_{6}$) |  |  |  |  |
| Coefficient | 0.024 | 0.055** | 0.120*** | 0.075*** |
| 95% CI | [-0.021, 0.069] | [0.004, 0.107] | [0.048, 0.191] | [0.026, 0.123] |
| Effect on trend of intervention ($\beta_{7}$) |  |  |  |  |
| Coefficient | -0.005*** | -0.007*** | -0.006*** | -0.006*** |
| 95% CI | [-0.006,-0.003] | [-0.008,-0.005] | [-0.009,-0.004] | [-0.008,-0.004] |

*** p<0.01; ** p<0.05; * p<0.1

**Methodological Annexure**

Interpretation of parameters of ITSA with control group

The parameter $\boldsymbol{\beta}_{\boldsymbol{0}}$ is the control group’s level in the first month (January 2007); $\boldsymbol{\beta}_{\boldsymbol{1}}$ is the pre-intervention slope for the control group; $\boldsymbol{\beta}_{\boldsymbol{2}}$ is the difference in the control group's level between the pre and post-intervention periods; $\boldsymbol{\beta}_{\boldsymbol{3}}$ is the difference in the control group's slope between the pre and post-intervention periods; $\boldsymbol{\beta}_{\boldsymbol{4}}$ is the difference in level between the treated and control groups in the first month (January 2007); $\boldsymbol{\beta}_{\boldsymbol{5}}$ is the difference in the pre-intervention curve between the treated and control groups; $\boldsymbol{\beta}_{\boldsymbol{6}}$ is the difference in level between the pre and post-intervention periods and between the treated and control groups; and $\boldsymbol{\beta}_{\boldsymbol{7}}$ is the difference in slope between the pre and post-intervention periods and between the treated and control groups.

Synthetic control group and donor pool of diseases

Synthetic control is a data-driven procedure. To construct the control group, units belonging to the donor pool are combined to obtain a level and trend comparable to the treated unit group. The donor pool is a group of potential control units, such as non-smoking-related diseases or diseases not included in the GES program. Therefore, some units are selected to construct the synthetic control group. The ponderation between donor pool units is so that the synthetic control group replicates the pre-intervention period performance of the treated unit, considering the trend and level of the series. Then, it could be assumed that this synthetic control group constructed performance is a counterfactual of the post-intervention treated unit performance.

In this study, the methodology used to construct the synthetic control group is SCUL (Synthetic Control Using LASSO), a method developed by Hollingsworth and Wing [1]. The SCUL method uses penalized ordinary least squares (OLS) to correct the over-adjustment that the time series autocorrelation could generate. Over-adjustment could lead to poor off-sample predictions [1].

The list of diseases/conditions used as donor pool for the synthetic control groups contains diseases/conditions that are not related to either tobacco consumption or were covered by the GES program (the Explicit Health Guarantees program that was implemented nationally and implied improved and priority care for specific pathologies). These diseases/conditions are (in parenthesis is the International Statistical Classification of Diseases and Related Health Problems, ICD-10):

| 1. Intestinal infectious diseases (A00–A09) |
| --- |
| 1. Other bacterial diseases (A30–A49) |
| 1. Infections with a predominantly sexual mode of transmission (A50–A64) and Other diseases caused by chlamydiae (A70–A74) |
| 1. Viral infections of the central nervous system (A80–A89) |
| 1. Viral infections characterized by skin and mucous membrane lesions (B00–B09) |
| 1. Mycoses (B35–B49) |
| 1. Protozoal diseases (B50–B64) (minus B54 unespecified malaria) |
| 1. Disorders of thyroid gland (E00–E07) |
| 1. Disorders of other endocrine glands (E20–E35) |
| 1. Metabolic disorders (E70–E90) |
| 1. Organic, including symptomatic, mental disorders (F00–F09) |
| 1. Mental and behavioural disorders due to psychoactive substance use (F10–F19) (minus F17 Mental and behavioural disorders due to use of tobacco and F19 Mental and behavioural disorders due to multiple drug use and use of other psychoactive substances) |
| 1. Mental and behavioural disorders due to multiple drug use and use of other psychoactive substances (F19) |
| 1. Schizophrenia, schizotypal and delusional disorders (F20–F29), Mood [affective] disorders (F30–F39) and Neurotic, stress-related and somatoform disorders (F40–F48) |
| 1. Behavioural syndromes associated with physiological disturbances and physical factors (F50–F59) |
| 1. Disorders of adult personality and behaviour (F60–F69) |
| 1. Inflammatory diseases of the central nervous system (G00–G09) |
| 1. Systemic atrophies primarily affecting the central nervous system (G10–G14) (minus G14 Postpolio syndrome) |
| 1. Extrapyramidal and movement disorders (G20–G26) |
| 1. Other degenerative diseases of the nervous system (G30–G32) |
| 1. Episodic and paroxysmal disorders (G40–G47) |
| 1. Diseases of the eye and adnexa (H00–H59) |
| 1. Diseases of the ear and mastoid process (H60–H95) |
| 1. Diseases of appendix (K35–K37) |
| 1. Gastroenteritis (K52) |
| 1. Paralytic ileus and intestinal obstruction without hernia (K56) |
| 1. Cholecystitis(K81) |
| 1. Dermatitis and eczema (L20–L30) |
| 1. Papulosquamous disorders (L40–L45) |
| 1. Urticaria and erythema (L50–L54) |
| 1. Decubitus ulcer and pressure area (L89) |
| 1. Other soft tissue disorders (M70–M79) |
| 1. Calculus of kidney and ureter (N20) and Calculus of lower urinary tract (N21) |

*Placebo test*

When using Synthetic Control Method is recommended to perform placebo tests. These tests consist of finding a synthetic control group for every unit on the donor pool to compare the effect of the intervention on the treated units with units that should not be affected by the law. It is expected not to find any effect when evaluating the control units; then, it can be assured that the impact of the intervention is not related to an external factor.

One placebo test compares the intervention's effect on the treated unit to a distribution of the effect estimated of the intervention on the units of the donor pool. This comparison is made through a hypothesis test. The null hypothesis is that the marginal effect estimated for the treated unit is the same as that for the control units. Therefore, we want to reject the hypothesis. Tables A1 and A2 show the hypothesis tests’ p-values for hospitalizations and deaths.

Table A1: Hypothesis tests' results, treated unit effect on hospitalizations compared to the placebo effect

|  | Ischemic Heart Disease | | Acute Myocardial Infarction | | Stroke | |
| --- | --- | --- | --- | --- | --- | --- |
|  | Level | Trend | Level | Trend | Level | Trend |
| Entire Population | 0.2640 | 0.0471 | 0.0035 | 0.0000 | 0.3275 | 0.4097 |
| 20-44 years | 0.0000 | 0.0013 | 0.0005 | 0.0676 | 0.0001 | 0.0000 |
| 45-64 years | 0.0716 | 0.0000 | 0.2213 | 0.0000 | 0.0673 | 0.4442 |
| 65+ years | 0.0001 | 0.0002 | - | - | 0.2959 | 0.0138 |

Table A2: Hypothesis tests' results, treated unit effect on deaths compared to the placebo effect

|  | Ischemic Heart Disease | | Acute Myocardial Infarction | | Stroke | |
| --- | --- | --- | --- | --- | --- | --- |
|  | Level | Trend | Level | Trend | Level | Trend |
| Entire Population | 0.0487 | 0.0026 | 0.0181 | 0.0000 | 0.4812 | 0.0092 |
| 20-44 years | 0.0002 | 0.0000 | 0.0000 | 0.0001 | 0.0477 | 0.0000 |
| 45-64 years | 0.0000 | 0.0000 | 0.0518 | 0.0000 | 0.0001 | 0.0000 |
| 65+ years | 0.0039 | 0.0248 | 0.0001 | 0.0000 | 0.0389 | 0.0072 |

Figures A1 and A2 show the differences between the treated unit and synthetic control group for each donor pool’s units for hospitalizations and deaths. Again, it can be seen that there is no predominant pattern in any direction, showing that there is no temporal explanation for the effect found in the treated units.

Figure A1: Difference between treated unit and synthetic control group for placebo test on hospitalizations


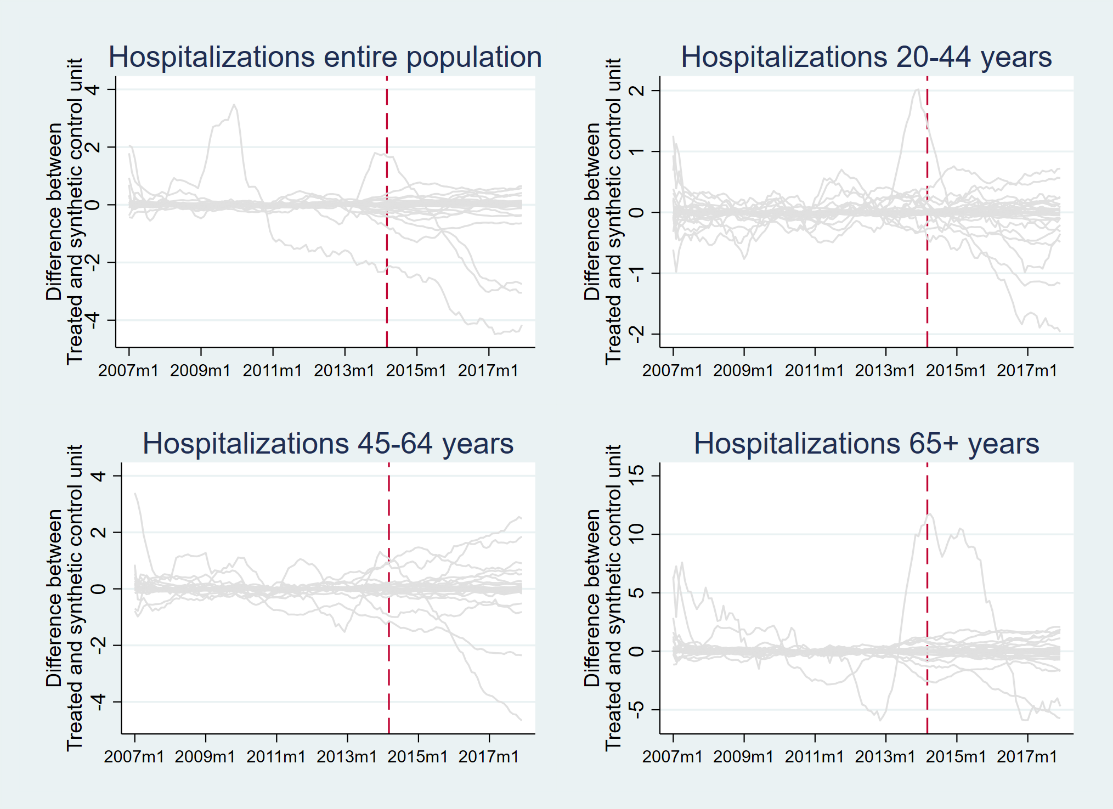


Figure A2: Difference between treated unit and synthetic control group for placebo test on deaths


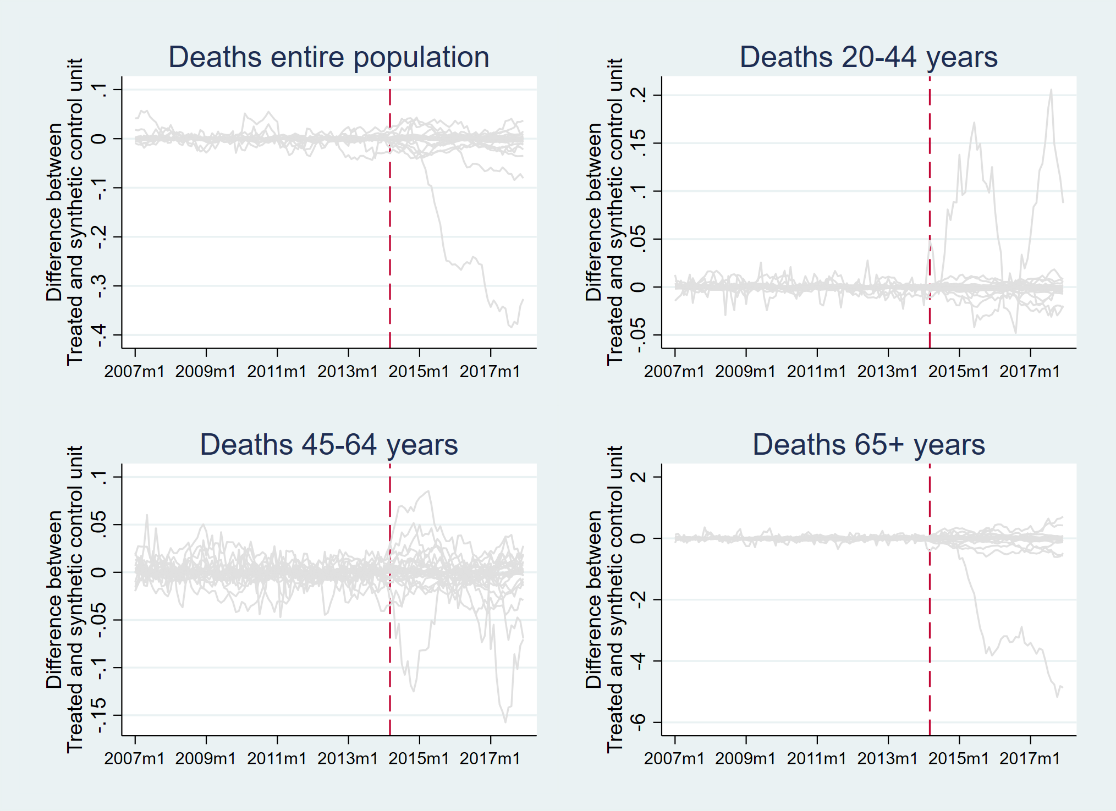


**References**

1. Hollingsworth A, Wing C. Tactics for design and inference in synthetic control studies: An applied example using high-dimensional data. Available at SSRN 3592088 2020.
